# Supplementary material for: Function-specific epistasis shapes evolutionary trajectories towards antibiotic resistance
Source: Nat Commun. 2026 Jul 28;17:7564. doi: 10.1038/s41467-026-76025-1 (PMC13415927; doi:10.1038/s41467-026-76025-1)
Supplement: Supplementary file 1 — Supplementary Information [file 41467_2026_76025_MOESM1_ESM.pdf]

Supporting Information

Function-specific epistasis shapes evolutionary trajectories towards antibiotic resistance

Gabriela Petrungaro[1,‡], Theresa Fink[1], Booshini Fernando[1], Gerrit Ansmann[1] and Tobias Bollenbach[1,2,‡]

1. Institute for Biological Physics, University of Cologne, 50931 Cologne, Germany
2. Center for Data and Simulation Science, University of Cologne, 50931 Cologne,

[‡] Correspondence: t.bollenbach@uni-koeln.de, gpetrung@uni-koeln.de

Supplementary figures

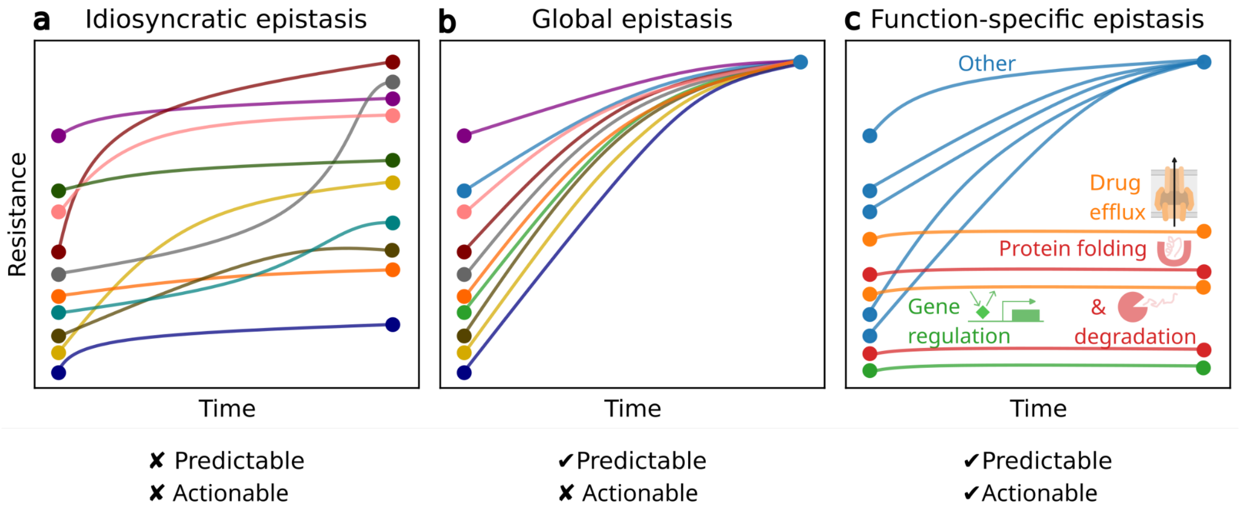

Supplementary Fig. 1: Schematic comparison of the different types of epistasis mentioned in this study. Unlike global epistasis patterns, where evolutionary increases in resistance can be predicted based solely on initial resistance values, or idiosyncratic epistasis, where evolutionary outcomes are unpredictable, function-specific epistasis enables partial predictions of resistance evolution based on the functions of disrupted genes. The identified functions may inform targeted actions to improve drug treatments using small-molecule inhibitors.

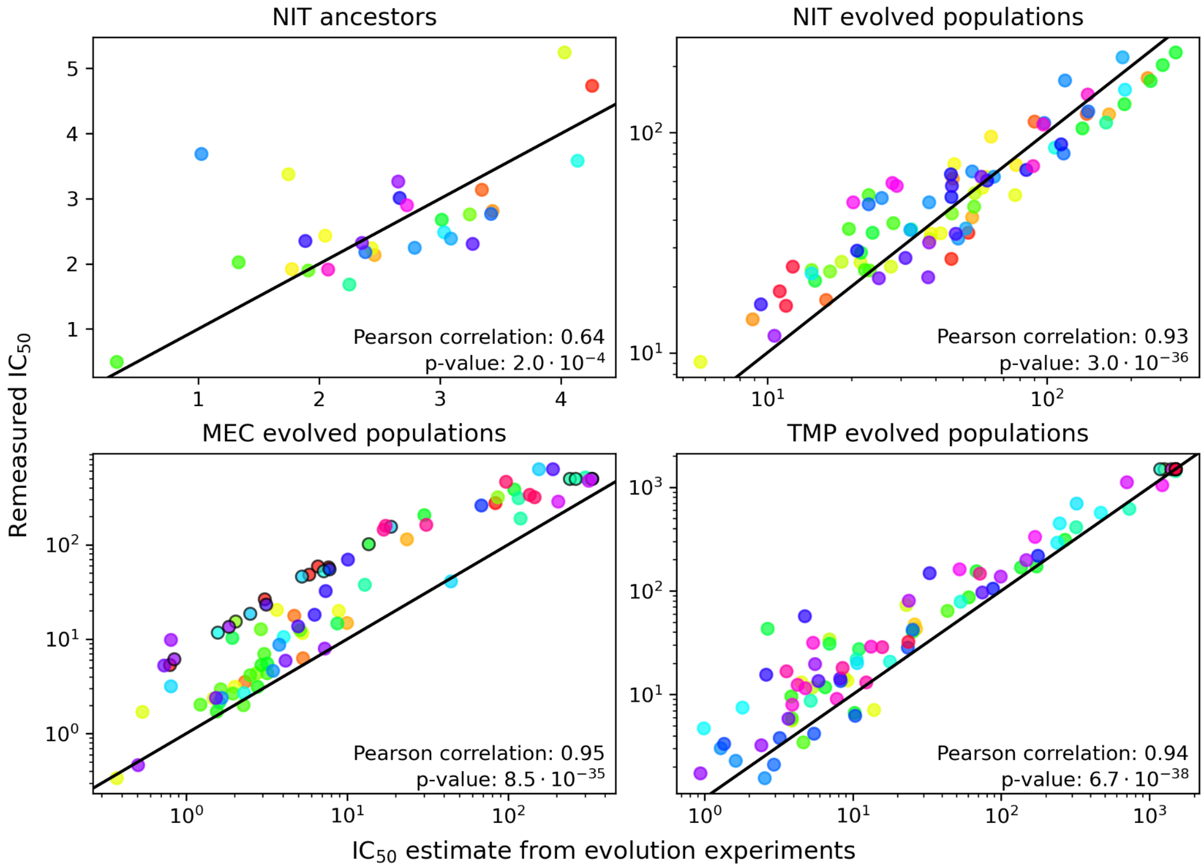

Supplementary Fig. 2: Comparison of IC_{50} values estimated from the evolution experiment with the values remeasured in dose-response curves after the experiment. The data are from experiment NIT_evo (Table 1). Due to the smaller data range, the IC_{50} values of the ancestors are shown on a linear scale. The black lines are the identity. Colors represent different gene-deletion strains. Circles with black edges show the maximum concentration used in the gradient for the dose-response curve measurement and represent a lower limit for the IC_{50}. These cases were excluded from the correlation analysis.

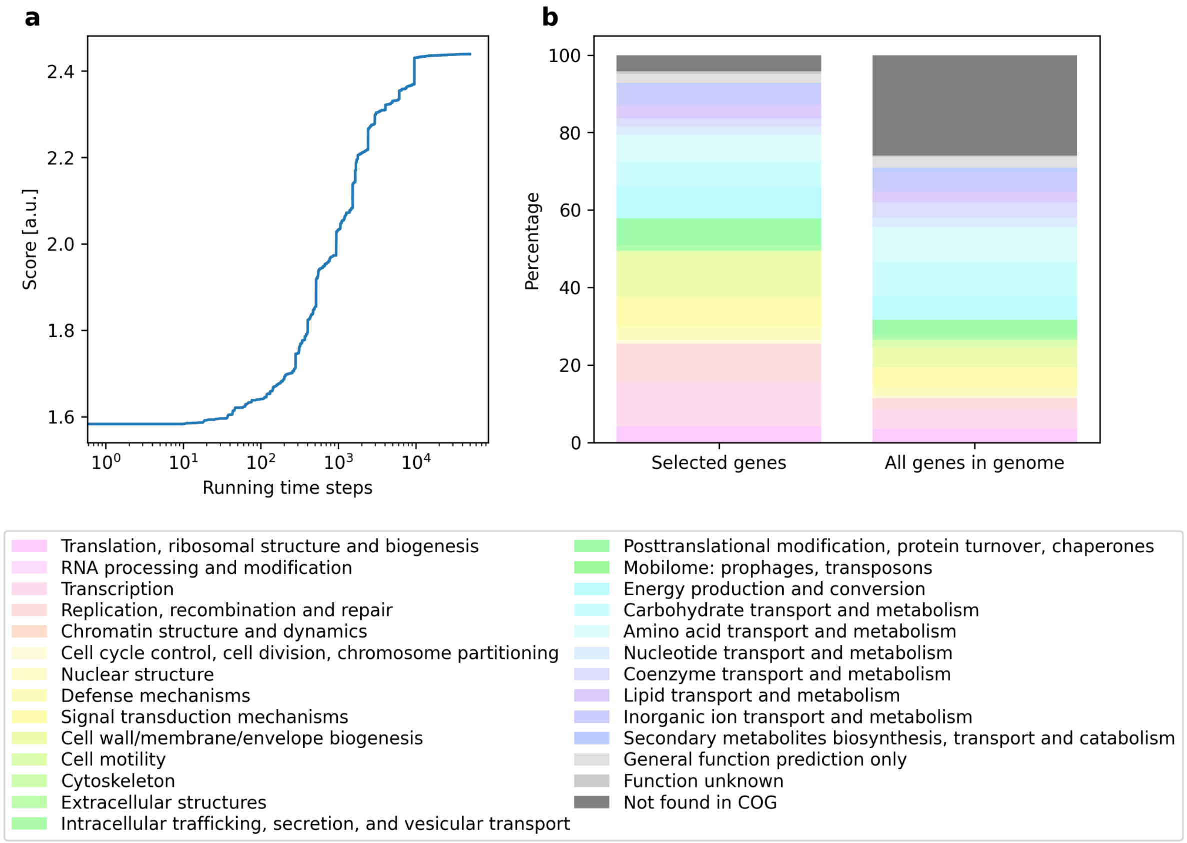

Supplementary Fig. 3: Functional annotation of selected gene-deletion strains. (a) Time evolution of the recursively increasing score based on the standard deviation of initial resistance levels for the three antibiotics considered in this study (Methods, section *Selection of gene-deletion strains for evolution experiments*). **(b)** Distribution of COG (Clusters of Orthologous Genes) for the selected genes, compared to the distribution of all genes in the genome.

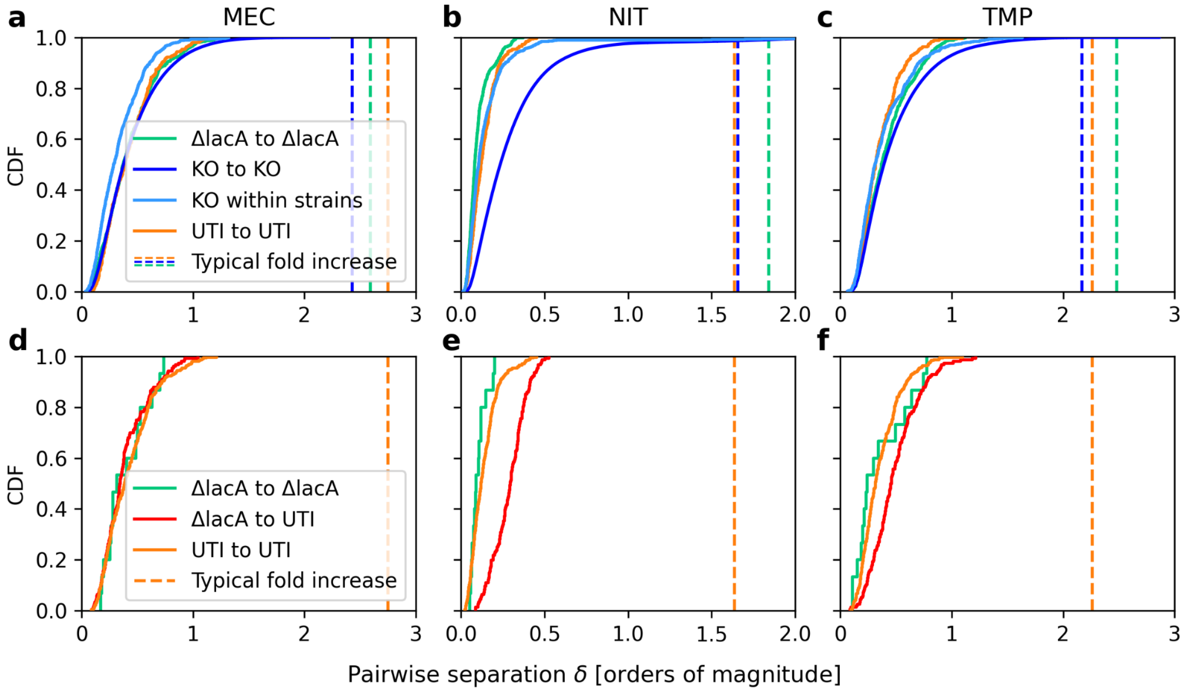

Supplementary Fig. 4: Distributions of the pairwise separation between resistance trajectories. (a–c) Pairwise separations δ between IC_{50} time series of gene-deletion strains (KO), either for all pairs (blue) or grouped by trajectories within populations that evolved from the same ancestral gene-deletion strain (light blue, weighted to give each strain an equal total weight), reference strain (*ΔlacA*; green) and clinical isolates (UTI; orange), respectively (Methods). Note that the sample size for the UTIs is too small for a meaningful within-strain distribution. The vertical lines show the median fold increase for the relevant strains (UTI isolates or gene-deletion strains, respectively) over the total duration of the experiment. Almost all values are far below these fold increases, showing that the phenotypic variability is generally much smaller than the typical resistance increase during evolution. Permutation tests show that the distributions for gene-deletion strains and UTI isolates significantly differ for NIT (p<0.001) but not for MEC and TMP (p=0.68 and p=0.051, respectively; Methods). For each antibiotic, the distributions of separations δ between all gene-deletion strains strains (blue) differ significantly (p<0.001, Methods) from those within gene-deletion strains (light blue). **(d–f)** Pairwise separation between UTI and reference *ΔlacA* strains (red) and between trajectories within these groups (UTI, orange; *ΔlacA*, green). For MEC, the *ΔlacA* trajectories are as close to the UTI trajectories as the UTI trajectories are to each other (permutation test yielded indistinguishable red and orange distributions, p=0.33). For NIT and TMP, trajectories of UTI strains and the reference strain are closer within these groups (orange and green) than across the two groups (permutation tests orange to red, p<0.01). However, the difference is much smaller than the typical increase over time (vertical line).

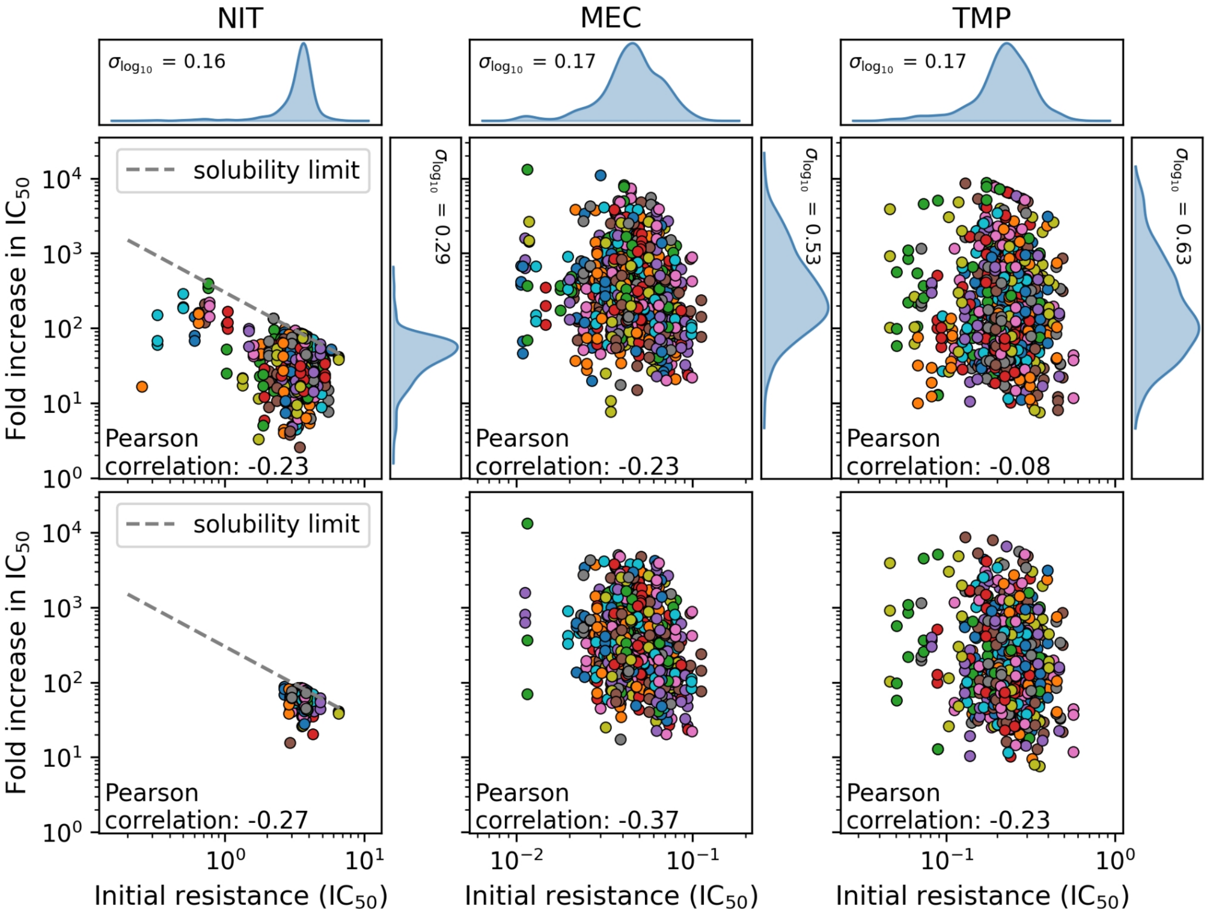

Supplementary Fig. 5: Diminishing returns epistasis does not adequately explain the increase in resistance. Upper panels: Fold-increase in resistance versus initial resistance (IC_{50}) for all populations (i.e., 259 gene-deletion strains including the reference strain) in the three evolution experiments NIT_evo, MEC_evo and TMP_evo (Table 1). Upper-most and right-most additional panels show kernel density estimates of the distributions of initial IC_{50} values and fold-increase in IC_{50} values, respectively. **Lower panels:** As upper panels, but excluding strains that significantly alter the evolution of resistance (significant gene deletions in Fig. 4 for each antibiotic). Pearson correlation was calculated based on the means of replicate populations from the same gene deletion to avoid pseudoreplication.

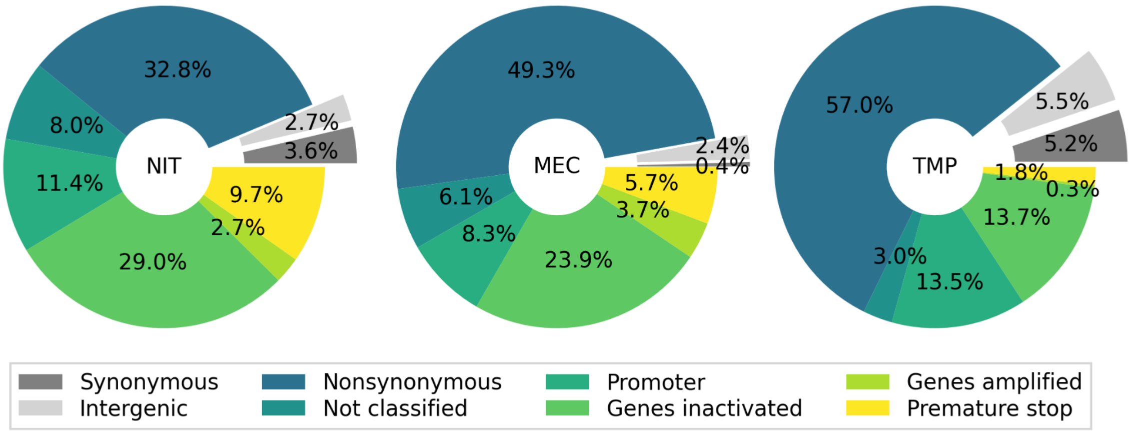

Supplementary Fig. 6: Fixed mutations in sequenced evolved samples. Comparison of the fractions of different mutation types from the total mutations called for each antibiotic. Mutator samples are excluded from this analysis. Intergenic and synonymous mutations (gray) were excluded from further analysis. See *Analysis of sequencing data* in Methods for details.

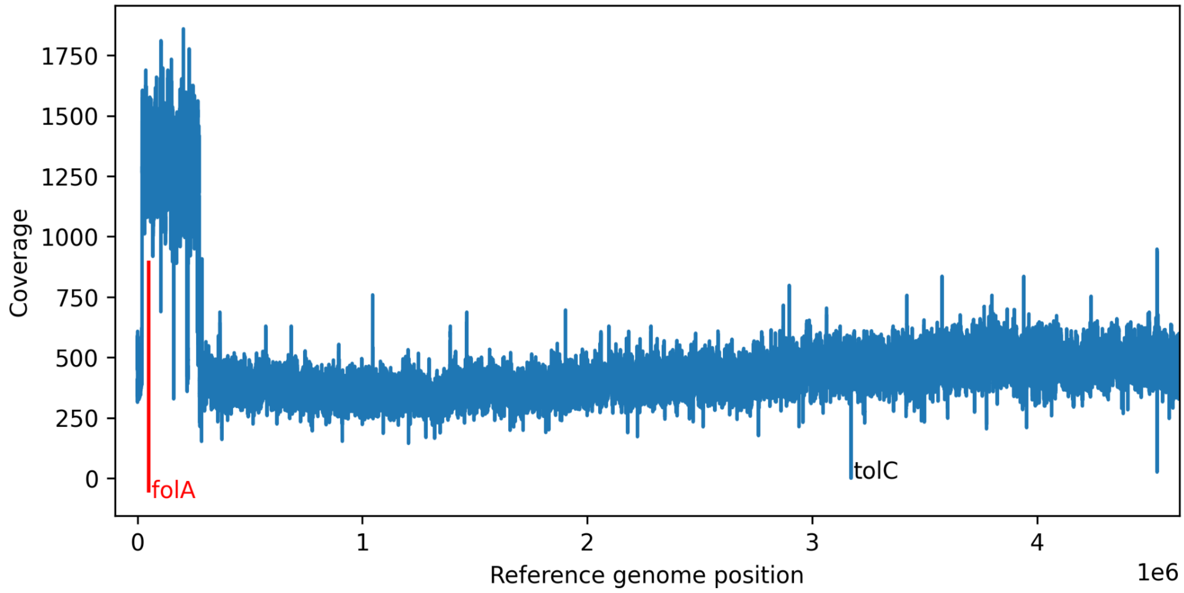

Supplementary Fig. 7: Example of sequencing coverage analysis. Data are from a *ΔtolC* strain that evolved in the presence of TMP (sample 11642_end_F9_tolC, Supplementary Data 1). A large amplification includes the *folA* gene, which encodes the target of TMP. See *Analysis of sequencing data* in Methods for details.

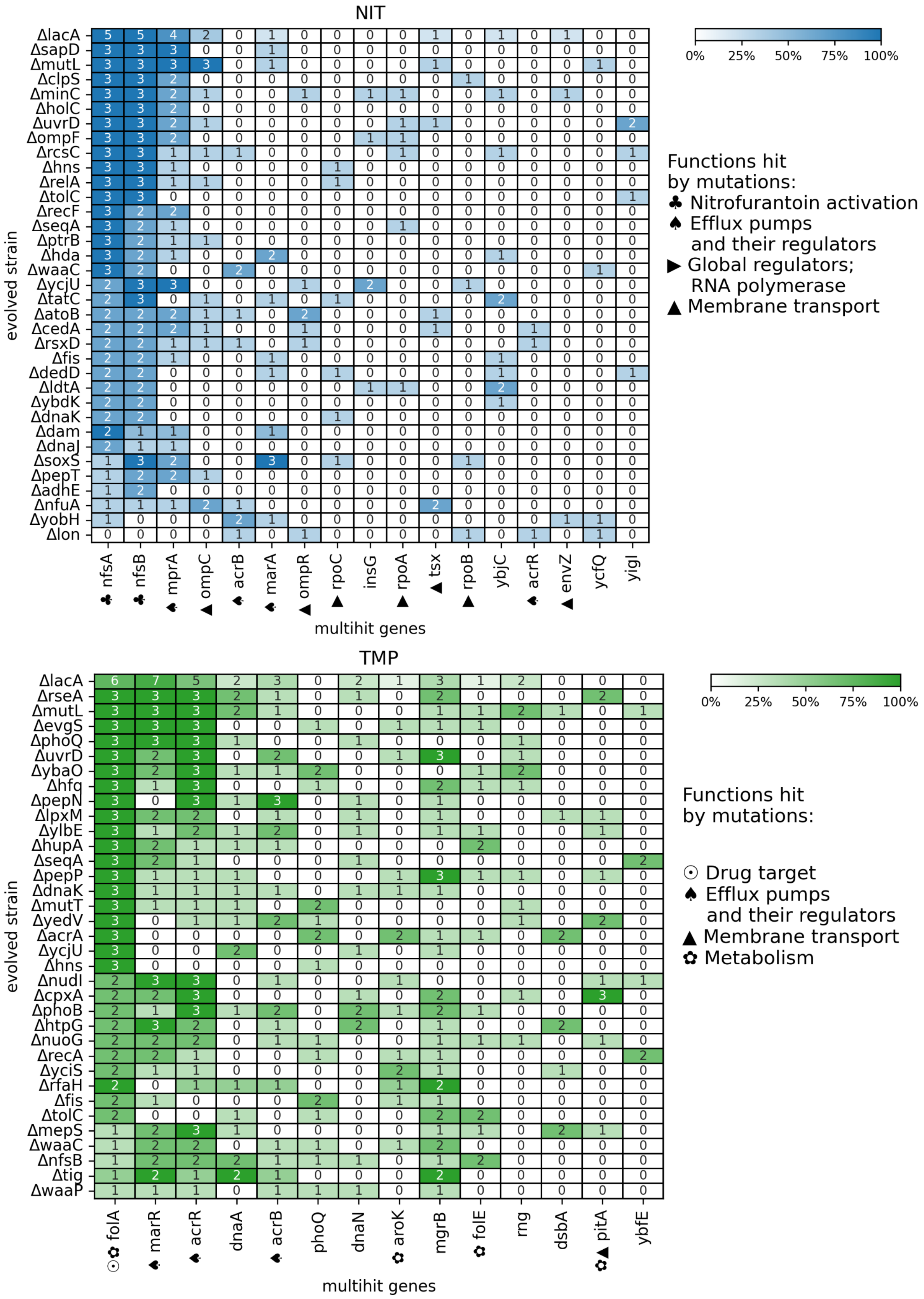

Supplementary Fig. 8: Overview of the mutations in multihit genes fixed in all sequenced evolved populations. As Fig. 3b, but showing the data for NIT (upper panel) and TMP (lower panel). Multihit genes are shown rank-ordered. Numbers and color intensity

indicate the number and percentage of replicate populations with the mutation, respectively. See *Analysis of sequencing data* in Methods for details.

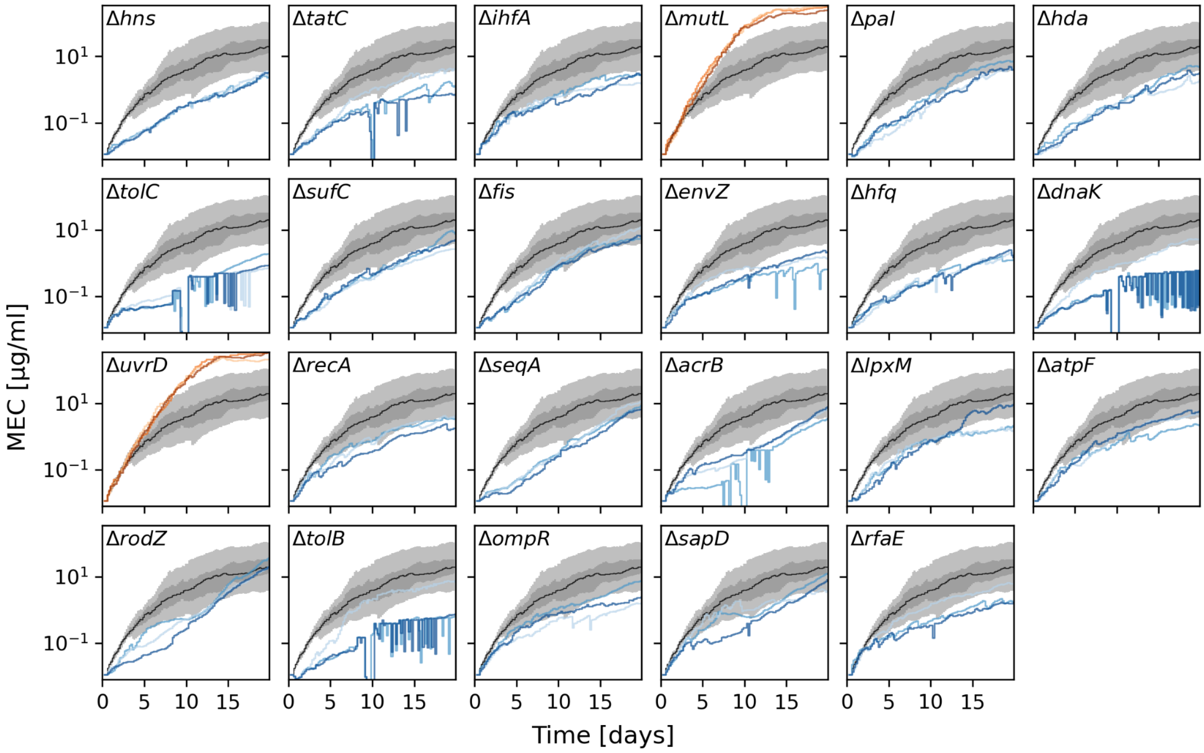

Supplementary Fig. 9: Gene deletions affecting resistance evolution to MEC. Estimated IC_{50} over time from the evolution experiment MEC_evo (Table 1). The dark and light gray shaded areas show the 50% and 90% ranges of IC_{50} values of the reference strain, respectively, and the black line indicates the median. The IC_{50} trajectories of specific gene deletions with significantly increased or decreased resistance evolution are shown in orange and blue, respectively. Different shades indicate three replicate populations, except for the gene-deletion strains listed in Supplementary Table 3, for which one or two populations died during the experiment.

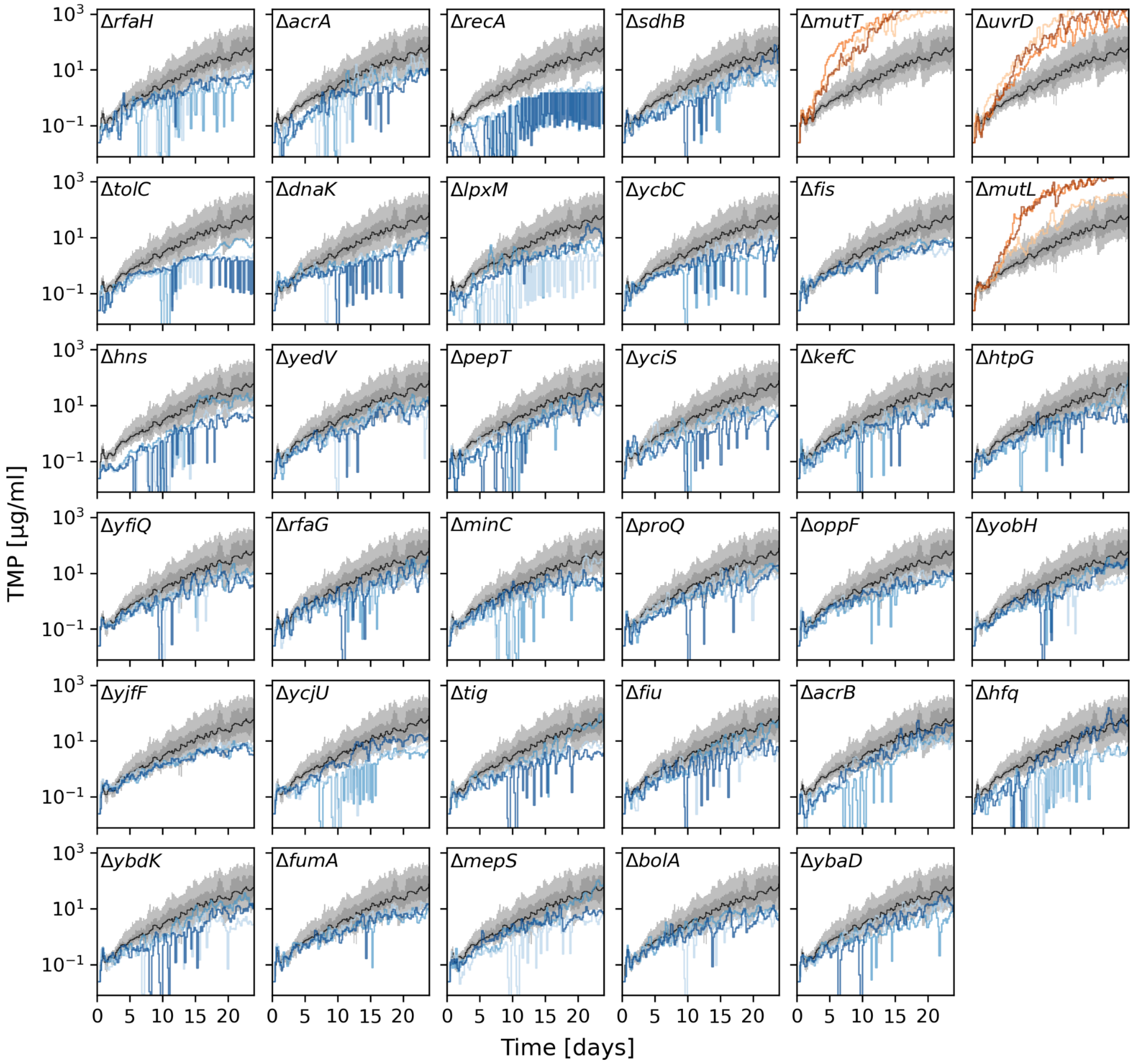

Supplementary Fig. 10: Gene deletions affecting resistance evolution to TMP. Estimated IC_{50} over time from the evolution experiment TMP_evo (Table 1). The dark and light gray shaded areas show the 50% and 90% ranges of IC_{50} values of the reference strain, respectively, and the black line indicates the median. The IC_{50} trajectories of specific gene deletions with significantly increased or decreased resistance evolution are shown in orange and blue, respectively. Different shades indicate three replicate populations, except for the gene-deletion strains listed in Supplementary Table 3, for which one or two populations died during the experiment.

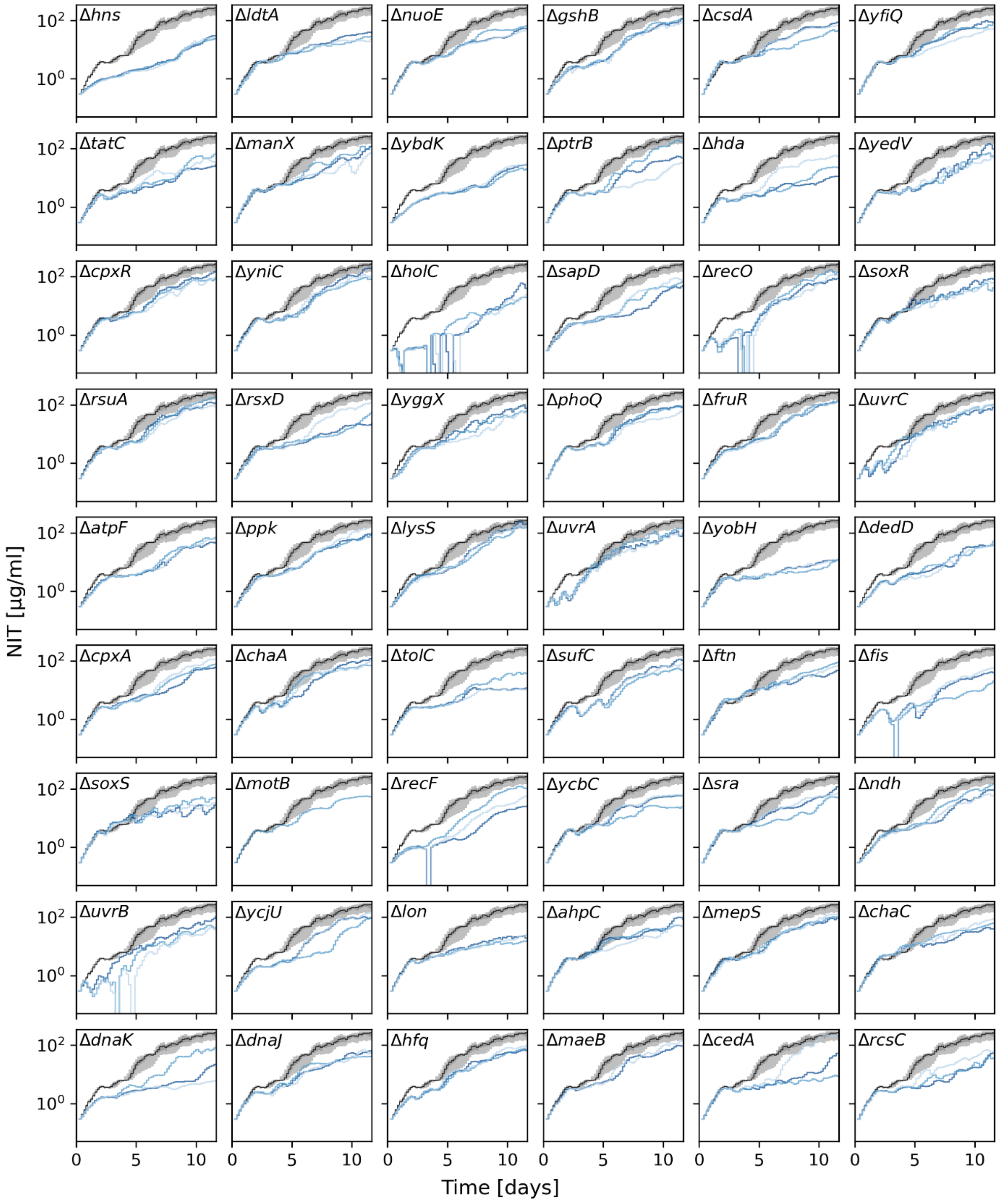

Supplementary Fig. 11: Gene deletions affecting resistance evolution to NIT (part 1 of 4). Estimated IC_{50} over time from the evolution experiment NIT_evo (Table 1). The dark and light gray shaded areas show the 50% and 90% ranges of IC_{50} values of the reference strain, respectively, and the black line indicates the median. The IC_{50} trajectories of specific gene deletions with significantly increased or decreased resistance evolution are shown in orange and blue, respectively. Different shades indicate three replicate populations, except for the gene-deletion strains listed in Supplementary Table 3, for which one or two populations died during the experiment.

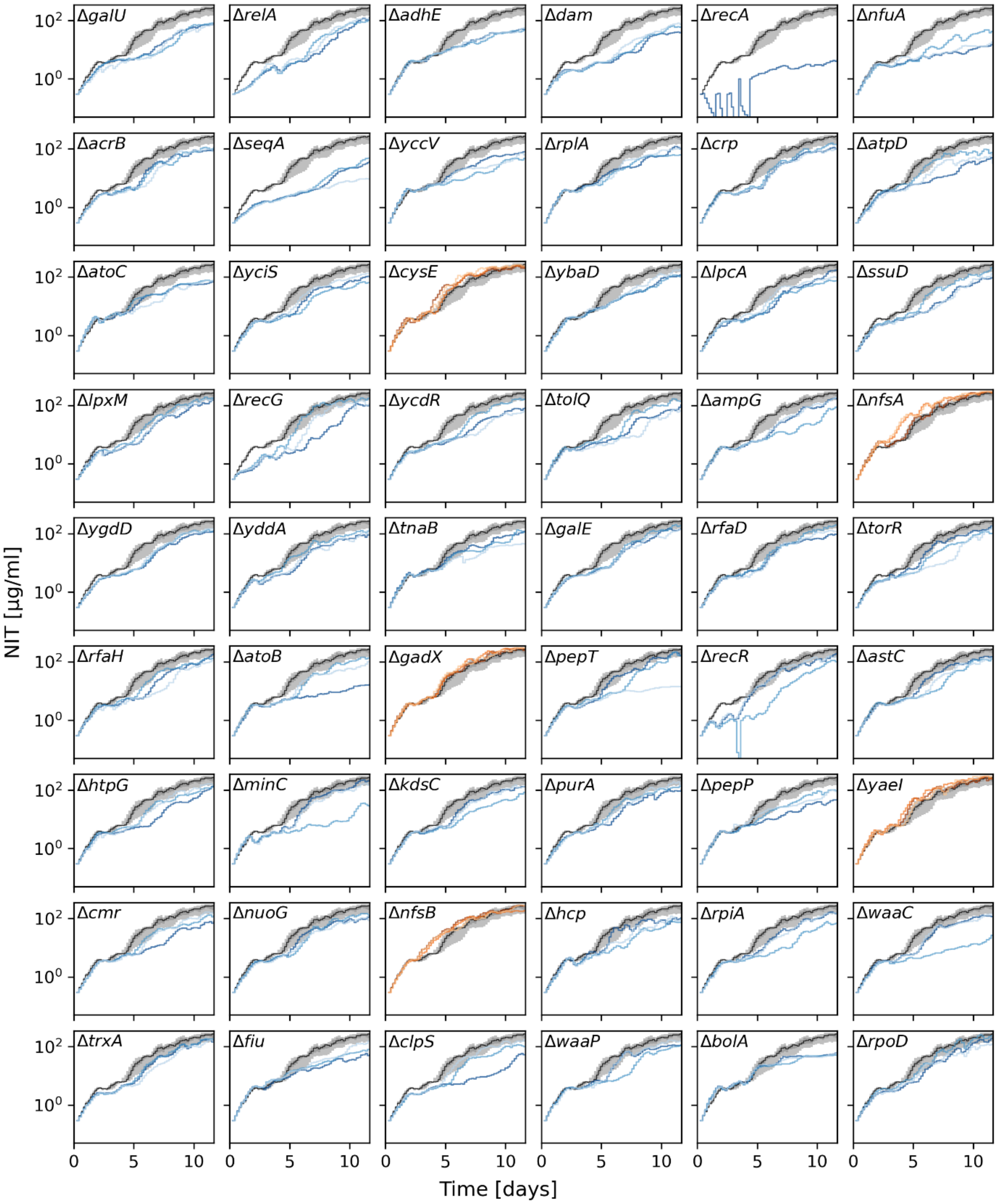

Supplementary Fig. 12: Gene deletions affecting resistance evolution to NIT (part 2 of 4). Estimated IC_{50} over time from the evolution experiment NIT_evo (Table 1). The dark and light gray shaded areas show the 50% and 90% ranges of IC_{50} values of the reference strain, respectively, and the black line indicates the median. The IC_{50} trajectories of specific gene deletions with significantly increased or decreased resistance evolution are shown in orange and blue, respectively. Different shades indicate three replicate populations, except for the gene-deletion strains listed in Supplementary Table 3, for which one or two populations died during the experiment.

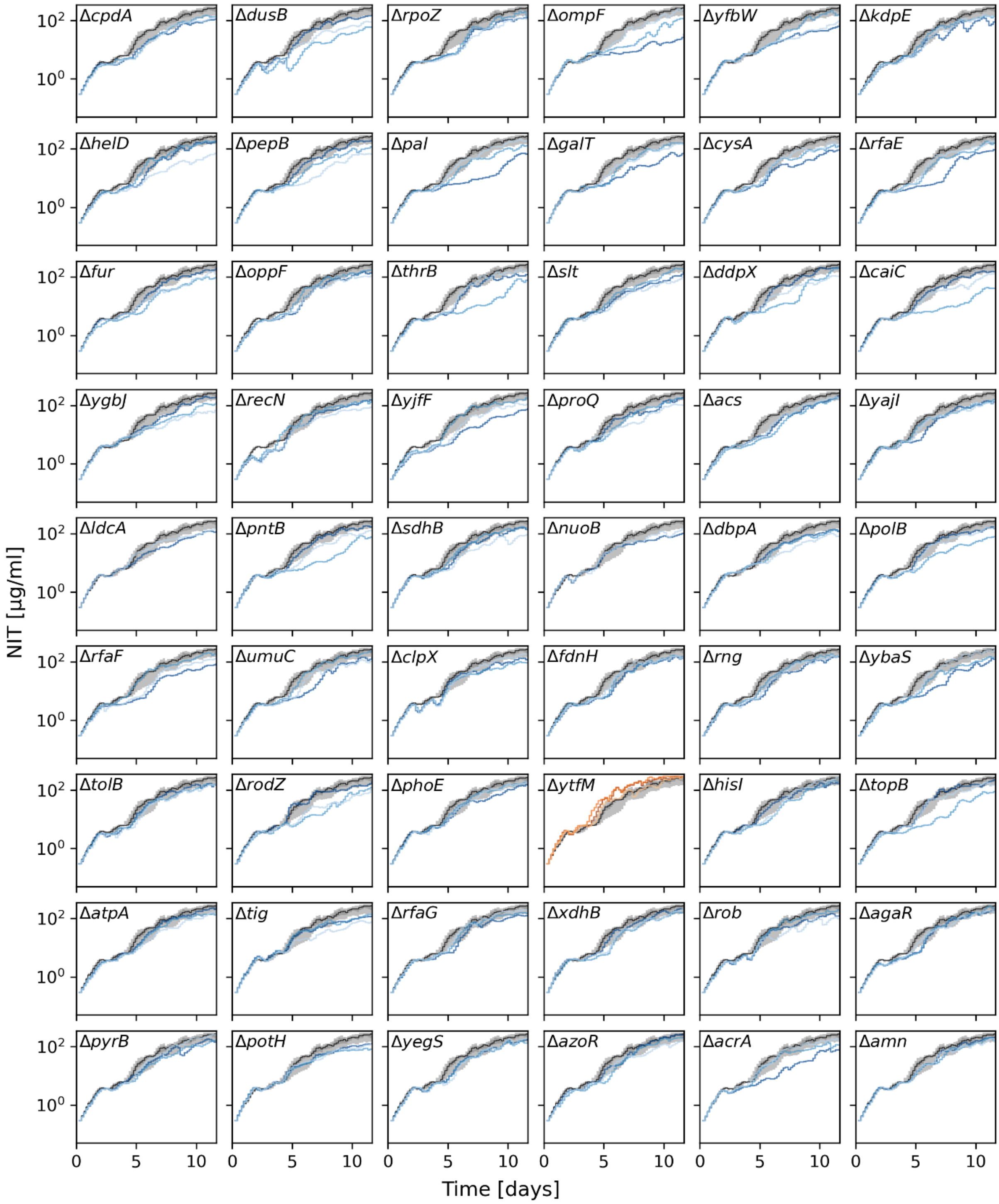

Supplementary Fig. 13: Gene deletions affecting resistance evolution to NIT (part 3 of 4). Estimated IC_{50} over time from the evolution experiment NIT_evo (Table 1). The dark and light gray shaded areas show the 50% and 90% ranges of IC_{50} values of the reference strain, respectively, and the black line indicates the median. The IC_{50} trajectories of specific gene deletions with significantly increased or decreased resistance evolution are shown in orange and blue, respectively. Different shades indicate three replicate populations, except for the gene-deletion strains listed in Supplementary Table 3, for which one or two populations died during the experiment.

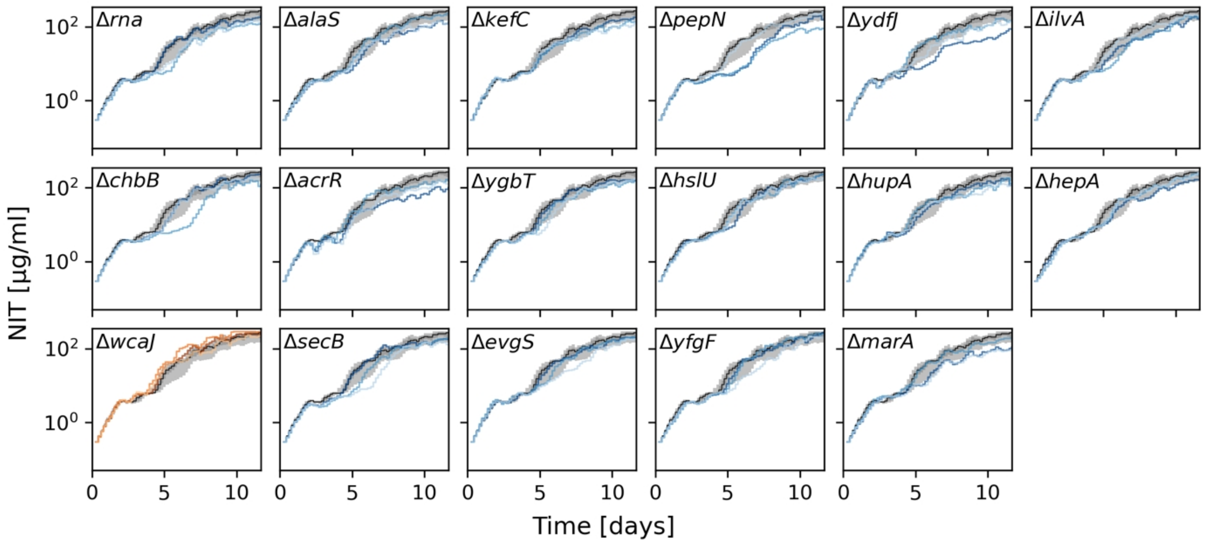

Supplementary Fig. 14: Gene deletions affecting resistance evolution to NIT (part 4 of 4). Estimated IC_{50} over time from the evolution experiment NIT_evo (Table 1). The dark and light gray shaded areas show the 50% and 90% ranges of IC_{50} values of the reference strain, respectively, and the black line indicates the median. The IC_{50} trajectories of specific gene deletions with significantly increased or decreased resistance evolution are shown in orange and blue, respectively. Different shades indicate three replicate populations, except for the gene-deletion strains listed in Supplementary Table 3, for which one or two populations died during the experiment.

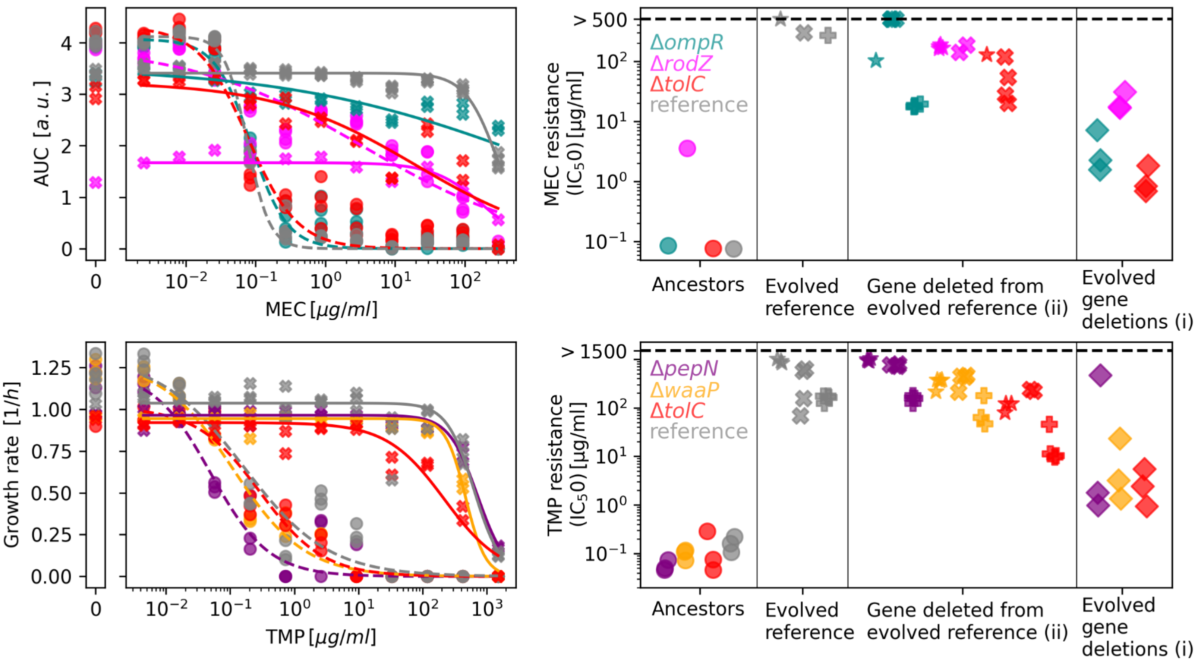

Supplementary Fig. 15: Comparison of (i) the extent to which lineages starting with a gene deletion evolve lower levels of resistance, versus (ii) the extent to which resistance drops when these same genes are deleted from a *ΔlacA*-evolved reference population. As Fig. 5g-h, but for MEC and TMP. (left panels) Examples of dose-response curves for ancestors (circles) and evolved reference strains (crosses) with and without one additional gene deletion. Repeated symbols show technical replicates used for the estimation of the dose-response parameters. (right panels) Comparison of the IC_{50} values obtained from left panels. Colors represent different gene deletions, and symbols indicate the evolved reference clones in which the gene deletions were introduced (Methods; mutations in the evolved reference populations in Supplementary Data 1). Black dashed lines indicate the maximum concentration used in the dose-response curve, which is the upper bound for the IC_{50} detection. Symbols and colors in the left panels match those in the right panels.

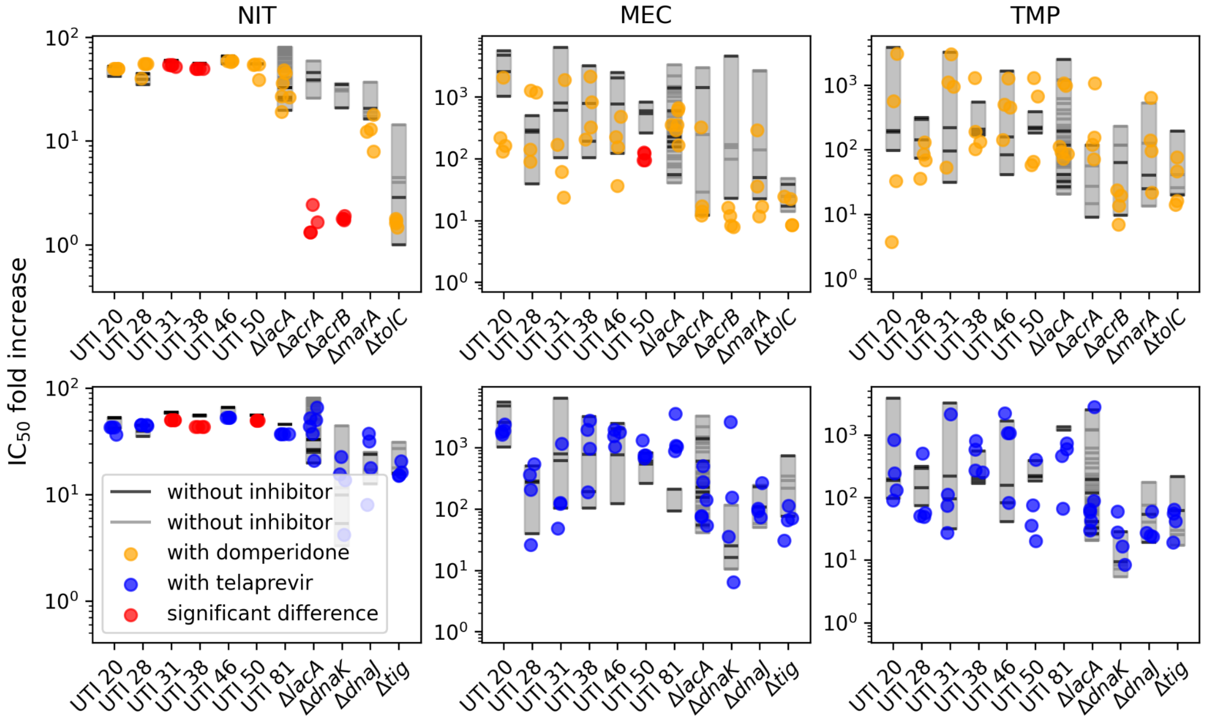

Supplementary Fig. 16: Complete set of comparisons of resistance evolution in the presence and absence of small-molecule inhibitors. The top row and orange points show the fold increase in resistance (IC_{50}) in the presence of domperidone (*cf.* Fig. 6d). The bottom row and blue points show the fold increase in resistance in the presence of telaprevir. Gray and black lines show replicates of the reference strain without inhibitor from two different evolution experiments (NIT/TMP/MEC_evo and inhibitors_evo, respectively, in Table 1). Gray rectangles show the range of fold increase for easier comparison. Red dots indicate strains where the inhibitor causes a significant reduction in fold increase (i.e. *p*-values <0.05 in two-sample *t*-tests using Benjamini–Hochberg false-discovery control). Note that in the case of NIT, significant differences except for *ΔacrA* and *ΔacrB* are artifacts because these strains reach the maximum NIT concentration with and without the inhibitor, but the initial IC_{50} changes due to the presence of the inhibitor.

Supplementary tables

Supplementary Data 1: Sequenced samples and their fixed mutations. Separate CSV file.

Sample	Number of mutations	Experiment
17226_19347_D5_clpS	40	MEC_evo
17221_19353_B1_acrA	37	MEC_evo
17221_19353_E3_seqA	19	MEC_evo
17222_19356_E3_seqA	17	MEC_evo
17223_19358_E3_seqA	16	MEC_evo
17225_19346_C2_pal	16	MEC_evo
11646_end_E1_nudI	299	TMP_evo
11641_end_F5_phoB	250	TMP_evo
11646_end_G5_ylbE	218	TMP_evo
11644_end_C5_lacA	150	TMP_evo
11645_end_A12_evgS	149	TMP_evo
11644_end_F10_pepP	127	TMP_evo
11647_end_H7_rseA	114	TMP_evo
11649_end_H7_rseA	93	TMP_evo
11641_end_G7_ybaO	80	TMP_evo
11641_end_H2_hupA	57	TMP_evo
11641_end_F9_tolC	37	TMP_evo
11643_end_G7_ybaO	32	TMP_evo
11645_end_C5_lacA	26	TMP_evo
11649_end_C2_lpxM	25	TMP_evo

Supplementary Table 1: Samples that spontaneously developed a mutator phenotype during evolution. The last part of the sample labeling indicates the deleted gene.

Experiment	Sample	Problem	Excluded?
NIT_evo	8962_830_B11_dam	cross-contaminated, another gene-deletion present in missing coverage	yes
TMP_evo	11649_end_B6_rfaH	contamination, no missing coverage at the expected gene position	yes
TMP_evo	11642_end_G4_tig	<10% of median coverage at the expected gene position and expected junction with KAN cassette not present in Breseq output	yes
TMP_evo	11646_end_F8_uvrD	<10% of median coverage at the expected gene position but expected junction with KAN cassette present	no

Supplementary Table 2: Samples with unexpected sequencing coverage in the gene-deletion loci.

Gene deletion	Number of dead populations	Experiment
recA	2	NIT_evo
motB	2	NIT_evo
nuoB	1	NIT_evo
maeB	1	NIT_evo
ldcA	1	NIT_evo
moaA	1	TMP_evo
sodB	1	MEC_evo

Supplementary Table 3: Populations that died during the evolution experiment and were excluded.

Primer	Sequence 5’ to 3’
upstream-lacA_Frw	ggttccttactggcattg
downstream-lacA_Rev	gctggaagtggttattctg
upstream-tolC_Frw	cgcgctaaatactgcttc
downstream-tolC_Rev	gttgccttacgttcagacg
upstream_*lon*_Frw	gtcatctgattacctggcgg
downstream_*lon*_Rev	cccgaattagcctgccag
upstream_*yobH*_Frw	gcgcatattgccgctacg
downstream_*yobH*_Rev	gagagtaagaacctgtcggaatatc
upstream_*rodZ*_Frw	tctacgttgaattggagcctgg
downstream_*rodZ*_Rev	gatggttcaccggcatctc
upstream_*ompR*_Frw	gaacagcaaggtgacgatgag
downstream_*ompR*_Rev	gatttagctggtgacgaacg
upstream_*rfaP*_Frw	gaatcatttttaattcataatctgcaaccc
downstream_*rfaP*_Rev	gcttgccagaaaaagccg
upstream_pepN_Frw_v2	cctcgtacaggagcgtttag
downstream_pepN_Rev_v2	gaatctgaaactcgcctgag
upstream_nfsA_Frw	cacagctgatgaaccgtcc
downstream_nfsA_Rev	ctgtcagacctggtcaaaagc

Supplementary Table 4: Primers used to generate and verify the deletion of genes of interest in evolved *ΔlacA* clones.
