# Supplementary material for: Function-specific epistasis shapes evolutionary trajectories towards antibiotic resistance
Source: Nat Commun. 2026 Jul 28;17:7564. doi: 10.1038/s41467-026-76025-1 (PMC13415927; doi:10.1038/s41467-026-76025-1)
Supplement: Supplementary file 2 — Description of Additional Supplementary Files [file 41467_2026_76025_MOESM2_ESM.pdf]

Description of Additional Supplementary Files

File Name: Supplementary Data 1
Description: Fixed mutations in sequenced samples. The three evolved *ΔlacA* reference populations with symbols in Fig. 5d–f,h and Fig. S15 are indicated by their symbols in a dedicated column named *symbol in Fig. 5 and S15*.

File Name: Supplementary Data 2
Description: p-values after false discovery control for the significant strains from the two-sided Mann–Whitney *U* tests in Fig. 4 (gene deletions shown in Venn diagram; Methods).
