# Supplementary material for: Function-specific epistasis shapes evolutionary trajectories towards antibiotic resistance
Source: Nat Commun. 2026 Jul 28;17:7564. doi: 10.1038/s41467-026-76025-1 (PMC13415927; doi:10.1038/s41467-026-76025-1)
Supplement: Supplementary file 6 — Transparent Peer Review file [file 41467_2026_76025_MOESM6_ESM.pdf]

nature portfolio

Peer Review File

Function-specific epistasis shapes evolutionary trajectories towards antibiotic resistance

Corresponding Author: Professor Tobias Bollenbach

This file contains all reviewer reports in order by version, followed by all author rebuttals in order by version.

Version 0:

Reviewer comments:

Reviewer #1

(Remarks to the Author)
Review on Petrungaro et al. entitled “Function-specific epistasis shapes evolutionary trajectories towards antibiotic resistance”

Whether global or idiosyncratic epistasis exhibit a stronger effect on the evolutionary trajectory of antibiotic resistance evolution is still unexplored apart from a couple of limited scale research with small sample sizes. Petrungaro et al. take on this challenge by implementing an automated high-throughput morbidostat platform capable of maintaining 864 parallel evolving populations simultaneously. This impressive experimental framework allows the authors to systematically examine how diverse genetic backgrounds influence both phenotypic resistance gains and mutational pathways under sustained selection.
The authors find that neither global epistasis nor idiosyncratic epistasis alone can fully account for the observed resistance trajectories. While a predominant part of evolution is highly repeatable, both at the phenotypic and the genotypic level and across widely different E. coli strains, they also identify clear cases in which perturbation of specific cellular subsystems constrains evolution into alternative, often less efficient, mutational trajectories. This “function-specific epistasis” is then explored in depth, and the manuscript provides several mechanistically compelling examples where disruption of efflux pumps, protein quality control, membrane biogenesis, or cell-shape maintenance reshapes the evolutionary landscape.
Overall, Petrungaro et al. present a carefully designed and thoroughly executed study that reveals how initial genotype, fitness, and resistance levels contribute to general trends in resistance evolution, while also uncovering the critical role of specific cellular functions in constraining or redirecting evolutionary paths. These findings substantially advance our understanding of antibiotic resistance evolution and will stimulate further research in both fundamental evolution and therapeutic intervention.

Suggestions for Improvement:

Throughout the text, it may be misleading that on the one hand the authors emphasize that “overall resistance evolution is highly repeatable” across genetically distinct E. coli strains, while in other parts they state that “genetic background strongly influences resistance evolution.” It would be helpful to rephrase these points to distinguish clearly between:
a general, global trend of high repeatability, and
specific, function-linked deviations affecting a minority of genetic backgrounds.
Explicitly emphasizing this distinction in the Abstract and Results would improve clarity.

Several gene deletions that strongly alter resistance evolution (e.g., tolC, lon, dnaK, rodZ) have broad cellular roles, and their effects may involve general physiological constraints rather than epistasis with specific resistance mutations alone. Even though ancestral growth defects were minimized, some deletions may limit metabolic flexibility or stress tolerance, thereby reducing access to certain mutational paths or imposing costs on alternative routes. A brief discussion acknowledging these potential fitness trade-offs would help clarify whether slowed evolution reflects direct genetic interactions or more global limitations on adaptability. This is especially relevant for “anti-evolution drug” implications.

For nitrofurantoin, several populations approach the maximum soluble concentration during evolution. As this ceiling artificially compresses late-stage variation, the authors could explicitly mention how this affects the interpretation of the relatively deterministic NIT trajectories.

Given the scale of the dataset and the complexity of the statistical analyses, making the raw IC_{50} trajectories and analysis scripts easily accessible (GitHub) would further enhance reproducibility and transparency.

The selection procedure for the 258 KEIO deletions is detailed and methodologically sound, but only presented in the Methods. A short summary in the Results, highlighting how this set was chosen, would help readers understand the rationale behind the experimental design.

Figures 3 and S9 present rich mutational data. The authors might consider adding a brief textual summary grouping recurrent hits into functional categories (efflux regulation, target modification, LPS biosynthesis, oxidative stress), to make it more understandable for non-specialist readers.

On Figure 1A, the phrase “258 gene deletions, each 3 populations” is somewhat misleading. Using the same formulation as in the figure legend “258 gene-deletion strains in triplicate” would be clearer and more consistent.

A brief mention in the main text of the mutator-screening procedure and its outcome (number of samples excluded) described in the Methods would help readers understand how mutators were handled.

Even though ΔlacA is a straightforward reference strain, stating the rationale behind in the main text the same way as it is included in the methods, would make the experimental design more accessible to a general audience.

Reviewer #2

(Remarks to the Author)
In this work, Petrungaro and colleagues evolve hundreds of single-gene knockouts from the Keio collection under selection by three different antibiotics. In each antibiotic, they observe that most knockouts follow broadly similar resistance trajectories, but a subset consistently deviates: these strains show a markedly reduced ability to evolve high-level resistance and thus act as evolvability suppressors. Through genomic analysis, they identify a small set of knockouts (notably tolC, dnaK, recA) that behave as robust evolvability suppressors across all three antibiotic regimes. They further demonstrate that small-molecule inhibitors targeting some of these common suppressors (tolC, dnaK) can modestly slow resistance evolution. More strikingly, they find a strong synergistic effect when domperidone is tested in knockouts of either subunit of the major efflux pump AcrAB, suggesting that analogous combinations of inhibitors might meaningfully constrain evolutionary escape routes.

Besides the general story of identifying evolvability suppressors and their potential clinical applications, the study offers at least three other interesting insights: 1) Experiments with E. coli UTI clinical isolates mirror the main patterns, which is a very wellcome result as reinforces the idea that laboratory evolution in model strains can generate principles relevant to pathogenic relatives. 2) Endpoint resistance level is highly idiosyncratic to the identity of the starting knockout, and only weakly related to its initial resistance level (in other words, they report a weak role for global epistasis). 3) Negative epistasis appears not the be the common explanation for the evolvability supressors: strong incompatibility between knockout backgrounds and typical resistance mutations is clearly supported for tolC but not for the other deletions tested.

I like the authors’ methods and analyses, and I find the main takeaways genuinely relevant. However, I have several concerns about some of the claims and, more generally, about how certain parts of the text and figures are presented. In addition, the manuscript could have benefited from more engagement with prior literature in the field. These issues reduce readability and hinder a full appreciation of the significance of the work. Here are my concerns:

1) In the introduction, I missed a clearer account aimed at a general reader that the quest for suppressors of antibiotic resistance evolution by single-gene deletion is an active research topic. This line of work was pioneered by their own previous study (Lukačišinová 2020 Nat Comm; and not just “Recent work” as humbly stated in line 10 of page 2), but also by Horinouchi 2020 Sci Rep and Gifford 2018 Nat Ecol Evol. Although the authors cite these references, they do not clearly explain to a naïve reader how the present work inserts within and in what ways it advances the field.

2) The second claim in the Results is that phenotypic resistance trajectories are highly repeatable. While I agree that the observed resistance trajectories are characteristic for each antibiotic, I find it problematic to reconcile with their claim that the y-axis in Fig. 2b and 2c is logarithmic and that, for example at time 200, the range encompassing 90% of the data spans almost two orders of magnitude (can we can this "highly" repeatable?). Overall, I miss a more precise metric of repeatability. For instance: what is the average distance between two trajectories sampled at random in resistance units (Euclidian distance, to keep it simple)? How much smaller are these distances within a strain relative to across strains? And how similar are these distances in E. coli UTIs compared with the KO strains? Clear, intuitive numbers would make this section more compelling.

3) I really like Fig. 2a. It makes sense that the KO dataset is more variable and tends toward slightly lower evolved resistance, as these strains have fewer genetic options to “tinker” with. However, I missed an analogous plot for Fig. S12 (UTI strains). It's very interesting that these strains behave closer to the control lacA, perhaps because most polymorphisms they carry have been passed through the filter of natural selection in the wild, and are not as disruptive as some of the KOs. I would strongly suggest including the analagous plot for UTIs in the main figure, as this is an important takeaway from their study.

4) Page 10, lines 4–23. The authors argue that, unlike in many microbial evolution studies, global epistasis explains only a small fraction of genotype effects on resistance gains. I missed a clearer account of the relevant literature here, as this remains an active and open question: on the one hand, Card 2019 PLoS Biol, Horinouchi 2020 Sci Rep, and Jangir 2022 eLife (PMID: 35943060) do not observe global epistasis, whereas Lukačišinová 2020 Nat Comm and Sánchez-Maroto 2025 PLoS Genet (PMID: 40853985) do. We don't really know how to reconcile these differences. The authors gloss over this rich and recent literature with the broad statement that diminishing-returns epistasis is "typically not observable" in short antibiotic-resistance evolution experiments (Card, Horinouchi). But Horinouchi 2020 spans up to 192 hours, which is comparable to the current study and also to Lukačišinová 2020! In addition, Sánchez-Maroto 2025 is a two-step adaptive walk similar to Card 2019, yet they see the pattern while Card and cols do not. Please revise.

5) Beyond including a more accurate description of the debate described above, the authors are missing here a major opportunity. Kryazhimskiy 2014 Science famously shows that global epistasis diminishes with time. Given the richness of the authors dataset, I wonder what would happen if the authors reproduced Fig. S3 for shorter timescales, say 50, 100, 200, 400 hours. This analysis is straightforward. In addition, Sánchez-Maroto 2025 PLoS Genet reports that the global trend becomes much clearer after removing evolvability-suppressor lineages. What would happen here if the authors removed lineages that strongly deviate from the common trend? If global epistasis still failed to appear in earlier time points without evolvavily supressors, that would be a valuable result. As stated, this is a missed opportunity to make a deeper contribution at a fundamental level, and I encourage the authors to explore it.

6) Fig. 1e–g is difficult to interpret at first glance because the x- and y-axes do not share common ranges or scales. Using consistent ranges would immediately illustrate that patterns differ across treatments in both speed and magnitude of fitness gains. I also believe it would be useful to show explicitly that the NIT experiment was cut short due to solubility limits, this helps readers understand the experimental constraints (no shame in this!). Finally, please use more human readable units such as days instead of hours.

7) Page 5, lines 12–15: "Consistent with recent observations in strains from the E. coli Long-Term Evolution Experiment (19,42), the effects of genetic background on the evolvability of antibiotic resistance appear to be mostly idiosyncratic...". As noted in point 4, the authors should more broadly discuss the literature on idiosyncratic versus predictable patterns, beyond the two LTEE papers.

8) Page 6, line 48: "Deletions of components and regulators of multidrug efflux pumps ... drastically reduced resistance evolution for all three antibiotics tested here (Fig. 4; Fig. S4–S6), as well as for tetracycline and chloramphenicol (Lukačišinová)". I missed here mention and discussion of Horinouchi 2020 Sci Rep, which also identified robust evolvability suppressors across three antibiotics (cefixime, ciprofloxacin, chloramphenicol), though the genes differ (lysR, arcA, gutM, fur). If future work across multiple antibiotic classes is conducted, do the authors expect general rules for evolvability suppressors? Perhaps not universally, but possibly for antibiotics sharing resistance mechanisms or physiological targets?

9) Page 8, lines 17–36: This passage is difficult to follow; at times it comes accross as poorly written, with long sentences containing multiple clauses and ambiguous subjects. For example, the sentence beginning "we hypothesized that deleting the specific genes from evolved strains carrying those resistance mutations would reduce their resistance to levels similar to those of populations that evolved from ancestors with the specific gene deletion". It also contributes to the confusion that it seems that when the authors refer to "Fig. 3b; Fig. S9" in line 24, they actually mean Fig. 5 and Fig. S10. Even if so, the genes mentioned do not fully match what is shown in Fig. 5g and Fig. S10 (where is yobH?). Please revise and try to use shoter sentences.

10) Related to the work presented in Fig. 5g and 5h: the text should specify the genotype of the evolved reference strains from which those genes were deleted. With 36 replicates available, what guided the selection of these particular 3 lineages? The Methods describe how a clone was chosen for the selected ΔlacA evolved populations (the most typical dose–response curve), but nothing is said about the choice of these 3 lineages in the first place. It would be very interesting to know the resistance mutations these selected ΔlacA lineages accumulated. Had the authors chosen others, maybe lon and yobH KOs would have had more effect in the evolved lines?

11) Fig. 5d–f is not very clear. Why are there blank spaces in the heatmap? What do the "..." separators represent? If the point is to show that replicates from low-evolvability backgrounds tend to share uncommon mutations, why not illustrate one example (e.g., pepN), and compare its mutation profile with several ΔlacA endpoint isolates and with endpoint isolates from non-suppressor backgrounds that harbor the most repeatedly hit mutations?

12) Page 8, line 45: "Further elucidating the underlying molecular mechanisms can be difficult." I am not fully convinced by this. I will not insist, as the study already contains an enormous experimental effort... but testing the most common mutations specific to suppressor backgrounds (and absent from the general pool) in both the suppressor background and a ΔlacA background could reveal cases of positive epistasis (i.e., mutations only beneficial in the presence of the evolvability supressor KO).

Minor points

13) Page 6, regarding WGS: "For each antibiotic, the selection included 5 to 8 replicates of the reference strain and all three evolutionary replicates for each of at least 30 gene-deletion strains whose resistance trajectories clearly diverged from the

reference strain". Selecting strains that “clearly diverged” biases the sample toward less repeatability. I think this should be highlighted, as it makes the resulting parallel evolution all the more remarkable.

14) Page 8, line 3: “Notably, for NIT, disruption of the Lon protease consistently prevented...”. I think it would be neat to mention that loss-of-function mutations in lon were previously proposed to impose a hard limit on evolvability in a psychrophile adapting to elevated temperature (Toll-Riera 2022 Sci Adv; PMID: 35857489).

15) I really like the experiments shown in Fig. 5g and 5h, testing how deleting an evolvability suppressor affects resistance levels in evolved strains. However, I think the caption can more clearly explain that Fig. 5h compares: (i) how much lineages starting with a suppressor mutation achieve lower resistance during evolution, versus (ii) how much resistance drops when deleting these same genes from a ΔlacA-evolved population. It is a neat and insightful comparison but undersold, in my opinion.

16) Fig. 3: “Numbers and color intensity indicate the number and percentage of replicate populations with the mutation, respectively.” I do not see a legend for the percentages. Please include one.

Reviewer #3

(Remarks to the Author)

The authors present a compelling manuscript that addresses the important question of how epistatic interactions influence the evolution of antibiotic resistance. They perform high-throughput experiments to track resistance evolution over time for three different antibiotics, using hundreds of gene-knockout strains. They used this data to estimate the extent to which variation in resistance evolution is determined by genotype, a key finding. Furthermore, by sequencing a subset of the final evolved populations, they add substantial evidence and novel detail into how gene deletions influence a strain’s potential mutational space for antibiotic resistance. The experiments are explained clearly, the main text figures are well-made and intuitive to understand, and the results are compelling. However, in certain places I believe the findings do not fully support the authors ultimate conclusions. Additionally, a strange pattern of extreme variation is present in a subset of the supplementary data which is not adequately explained. If these problems are addressed, it would improve an already strong piece of work.

Major comments:
1. Sometimes I think complicated and potentially contradictory results are simplified too much in order to create a clearer or more impactful narrative. This is most obvious in the discussion.
a. P9, L41-45. I think the authors should be careful saying they identified specific functions that influence evolution, as they don't do any kind of functional enrichment analysis. It's probably fine for the "across antibiotics" functions, as they have multiple genes for each and the functions are all known to influence resistance for various antibiotics. But the second sentence saying certain functions only influence resistance for specific antibiotics feels like too big a claim for the data. In the results, only one gene each is mentioned for those functions, and the functions are not labelled in Fig. 4a.
b. P10, L1-2. Did the small molecule inhibitors partially recapitulate these effects? The authors didn't sequence the populations evolved in presence of inhibitors, so they don't know if it influenced resistance evolution on a genotypic level. And neither of the inhibitors (for any of the antibiotics) changed the final IC_{50} for the ΔlacA reference strain, which would be akin to recapitulating the effect of a gene deletion.
c. P10, L28-29. The authors don’t adequately back up the statement that the populations converged on the same resistance mutations, as they only looked at whether the same genes were mutated. Looking at the dataset, 94% of mutations only occurred in one population, and 3% only in two populations. If resistance mutations in most of the top gene targets are highly disruptive mutations, it makes sense the authors wouldn't see the exact same mutations. This should be acknowledged and more careful word choice is needed to make clear the authors only look at the genotypic repeatability of evolution at an individual gene level, not an individual mutation level.
d. Following the above point, the authors also sometimes use the term “mutational trajectories”, which slightly misrepresents the fact that they only have sequencing data for the final populations, not over time. This is particularly confusing because they use the term “resistance trajectories” to accurately describe the longitudinal data for IC50s.
2. What is causing the dramatic drops and spikes in the IC50 measurements over time, seen in the resistance trajectories (most prominently in Fig. S6 for TMP but also Fig. S5 for MEC)? The only mention of it is P7, L24-25 saying there’s high variability between replicates (not within replicates over time). Steps are taken to not show this in the main text figures by applying smoothing functions (methods: P18, L1-14), where these drops and spikes are described as “noise in the data”. But these are consistent variations of orders of magnitude. Why does this happen? Why does it happen more with specific antibiotics? The lack of explicit acknowledgement and clearer explanations raises questions about the validity of the underlying data.
3. Throughout the manuscript, but particularly in the introduction, more clarity and consistency is needed when discussing the different types of epistasis. A variety of terms - global epistasis, microscopic epistasis, diminishing-returns epistasis, idiosyncratic epistasis, and function-specific epistasis - are used at various points. “Global” and “diminishing-returns” seem to be used interchangeably, despite not being exactly the same. Is “function-specific” simply a subset of “idiosyncratic”? These terms need clearer definitions and if using fewer distinct terms is possible it should be preferred.
4. It’s stated in the methods that all the experiments were performed at 30°C rather than optimal temperature of 37°C for E. coli. Why was this choice made? It should be stated in the main text and justified in the methods. It does not make the results any less valuable, but more discussion of the potential consequences is needed – particularly given that clinical UTI strains are used, and that comparisons are frequently made to results from published studies that used 37°C. Some caveating of those comparisons should be included, as, for example, genes are differentially expressed between the two temperatures.

5. There are several places where it seems the wrong figure is cited, and many places where figures (particularly supplementary) are cited out of order (e.g. Fig. S12 cited before Fig. S3). I have pointed out several of these places in the minor comments, but the manuscript needs to be fully checked to ensure every figure citation is correct, and that figures are labelled in the order they appear in the main text.
6. Table S1 appears to be missing?

Minor comments:
1. P4, L23-26. The reference vs knockout distributions do not look similar to me. And the next sentence points out that they are different, as resistance gains are lower in knockout strains. Statistical tests are needed to determine if the difference between reference and knockout distributions is significant. If the distributions are significantly different, then the first sentence is hard to justify.
2. P4, L39. Assuming the authors have sequenced the genomes of the seven isolates used for this experiment. They should report on the % of genes shared between the strains.
3. P4, L41-44. The resistance trajectories do not seem remarkably similar for NIT in Fig. 2f, it looks like the isolates consistently evolved resistance faster. This leaves me extra confused by the p-values mentioned in the text, which say NIT is the least significant. Why are S12 and significance tests only looking at resistance levels at the end of the experiment, which misses the earlier-occurring resistance? Could the low different between ΔlacA and UTI isolates at the end of the experiment for NIT be related to the solubility limit?
4. P5, L1 Has this claim been shown or justified so far? No figure is cited. I guess the following figures explain it, but it’s a strong claim to not provide immediate evidence.
5. P5, L22. It's clear in the figure, but please make it clearer in the text that although initial resistance is a subset of initial genotype, it is not a very large subset. As is, talking about the high importance of initial genotype "...including initial resistance" feels contradictory to the previous paragraph about how initial resistance didn't matter much.
6. P6, L42. Fig. 4a has 186 total strains in the Venn diagram, out of 258 strains total. So this does not seem to be "rare"?
7. P7, L2. Fig. 4 as a whole is referenced here, but Fig. 4b-d are never specifically referenced. This is probably fine, as Fig. 4b-d are clearly just showing the IC_{50} curves for a subset of the data. But because they are not mentioned specifically in the main text, I am left with some questions:
a. why pick these specific three gene deletions? I see each fits into one of the categories you mention in L3-L4. But LPS biosynthesis category (lpxM) is not shown.
b. Also why only showing us gene deletions that evolved resistance more slowly? Would also be helpful to see ones that consistently evolved more quickly (e.g. mutL, uvrD).
c. More information about why these specific deletions were selected should be added briefly to figure legend, and explicitly in methods.
8. P7, L17. The authors give an example gene for every other functional category, but not response to DNA damage. Because Fig. 4a; Fig. S4 is referenced immediately after this text I looked for them there, but response to DNA damage is not mentioned in there either.
9. P7, L24-25. The greater divergence between replicates isn't obvious to me from Fig. 1g, as the variability metric is comparable to MEC in Fig. 1e. And in Fig. S4-6 it’s hard to visually compare replicates. Although I do see the high pattern of high variation (see major comment) is very present in Fig. S6.
10. P7, L26-28. This feels like an insufficient explanation for TMP being unique, given that Fig. 3a shows that TMP has the highest mutation diversity and that NIT mutations are also mostly in two mechanism-specific genes (nsfA/B).
11. P7, L32-33. This is an interesting result, but there's no figure cited to back it up. Fig. 3a shows that the same genes were mutated in most samples, but this doesn't tell me anything specifically about the deletion strains that had slowed resistance evolution. Are the most-mutated-genes more or less common in slow-evolution strains compared to other strains?
a. Also, be careful with the wording of "...carry a subset of the common beneficial mutations". This may well be true, but so far the authors have only shown that the populations share common genes that are mutated. The authors haven't shown if the specific mutations within those genes are the same.
12. P8, L26. What are the common resistance mutations?
13. P8, L28. Fig. 5g only shows data for NIT, although previous sentence says they did this for all antibiotics. Can the authors include the same dose-response curve plots for TMP and MEC in the supplementary and cite here with Fig. 5g.
14. P9, L22-26. This result is very surprising to me. If domperidone binds AcrB (as indicated by Fig. 6a and shown in ref 47), then why is it having an effect in a ΔacrB strain?
15. P10, L21-23. I'm not sure what this final sentence is doing, and it left me more confused. Do the authors consider this work a short evolution experiment? I'm assuming not, given that the authors appear to observe resistance gains approaching saturation. Even though (supposedly like a short evolution experiment) they don't observe diminishing returns epistasis. Are the authors saying that the fact that they didn't find it here is interesting, given it was a long enough experiment to detect it if it was present? If so, make this explicit.
16. P10, L28-29. the authors statement that the populations converged on the same resistance mutations is not well justified, as they only looked at whether the same genes were mutated. Looking at they dataset, 94% of mutations only occurred in one population, and 3% only in two populations. If resistance mutations in most of the top gene targets are highly disruptive mutations, it makes sense they wouldn't see the exact same mutations. But this should be acknowledged and more careful word choice is needed to make clear the authors only look at the genotypic repeatability of evolution at an individual gene level, not an individual mutation level.
17. P11, L12-14. So this sentence is saying, because the evolved reference population (with common mutations) often are not affected by knocking out the specific genes, then the effect of these genes on mutational paths often cannot be explained by epistasis? This sounds contradictory to the broader narrative that epistasis is responsible for the observed effects.
18. P11, L30-32. As I mentioned in minor comment 3, from Fig. 2f this really does not appear to be true for NIT. When that's 1/3 of the antibiotics tested, a blanket statement like this feels misleading.
19. P14, L24. Typo: "...the general increasing general trend."

20. P15, L9-12. If AUC has to be used for MEC, then it should also be used for the other antibiotics. The values are not really comparable otherwise.
21. P16, L22. Why is only data for NIT shown and not MEC or TMP?
22. Figure 1
a. for b-d caption: can you be explicit with what growth rate is normalized to. It's clear from methods, but I had to check what g/g0 meant.
b. Defining the population size as (∝ OD) and selection pressure as (∝ g/g0) confused me. This " ∝ " proportion symbol isn't used elsewhere in the manuscript, so can you just say "is proportional to"?
c. for e-g caption: It looks like dark grey is 50% of data and light grey is 90% of the data?
23. Table 1. There is no table legend. most columns are self-explanatory, but could benefit from an explanation of "steepness" and "maximum update factor". Also, what is exponent in the last row for this column? Also, column title says "Duration[days]" but units are given in hours.
24. Issues with figure citations:
a. P4, L30. Cites Fig. 2b but the sentence is about NIT, which is Fig 2c
b. P4, L44. Fig S12 is cited after Fig S2
c. P5, L23-25. Fig 2h is cited after Figure 2i-j
d. P6, L17. Fig S7 is cited after Fig S3
e. P6, L36. Fig. 3b is not mentioned or referenced until later (after Fig 4)
f. P6, L47. I think you also want to cite Fig S4 here (not just Fig S5-S6)
g. P7, L46. You mean to reference Fig. 5a-c here, as all antibiotics are mentioned. But also, they're mentioned in text in different order from their a-b-c order in the figure.
h. P8, L5. Pretty sure you mean to cite Fig. 5f instead of Fig. 5c
i. P8, L8. Again, should be Fig. 5e. And really for consistency with L5 which cites Fig. S9, here should also cite the Fig. 3b multi-hit table.
j. P11, L12. Think this is supposed to cite Fig. 5e-f. Aren't Fig. 5a-c about all the deletions as a whole and not specific strains?
k. P11, L14. If I understand what you're trying to say, you want to cite: (Fig. 5g-h, Fig. S10)
l. The caption for Fig. S1a references the wrong methods section (should be the first one about choosing gene deletions, not second one about choosing strains to sequence.

Version 1:

Reviewer comments:

Reviewer #1

(Remarks to the Author)
The revised manuscript by Petrungaro et al. shows clear improvement in clarity. The authors addressed the main concerns well. In particular, the difference between overall repeatability and specific genotype effects is now explained more clearly, which improves the message of the paper. The added discussion about possible general effects of some gene deletions is helpful and makes the interpretation more balanced. The clarification about nitrofurantoin and solubility limits also strengthens the results. Other changes, such as clearer figures and better explanations in the text, improve readability. Overall, the study is strong and well supported.

Reviewer #2

(Remarks to the Author)
I commend the authors for the substantial revisions made to the manuscript. The addition of new references, supporting figures (e.g., Fig. S1, Fig. S5), and expanded discussion has, in my view, addressed the major concerns raised during the first round of review by myself and the other reviewers. I now only have three minor comments, which I leave to the discretion of the authors and editor.

1) I appreciate the additional information on the functional categories of mutated genes provided in the revised Fig. 3. However, it appears that the diamond symbol is missing from the legend (envZ, ompR; presumably cell permeability?).

2) On the same Fig. 3, regarding the newly added functional categories, I would suggest reordering the columns and introducing small gaps to divide the heatmap into seven column blocks (corresponding to the six functional categories with multiple hits plus a seventh “other targets” category) while preserving the current row order. This would make certain patterns more readily apparent. For instance, mutations in the efflux category appear to accumulate within the same genetic backgrounds (i.e., multiple hits per lineage), whereas mutations in other categories tend to be mutually exclusive (e.g., cell shape: mrdA vs. mreB; cell wall: mrcB, mepM, nlpI, slt, prc). While a simple statistical analysis may be sufficient to support this observation (e.g. Fisher's test), I believe this point is important, as it directly informs the patterns of epistasis among the mutations acquired by each background.

3) Finally, I appreciate that the authors now provide a more rigorous analysis showing that the distributions of separations between all gene-deletion backgrounds differ significantly from those within gene-deletion backgrounds. However, I am less convinced by the current presentation in Fig. S4. As it stands, the figure emphasizes that phenotypic variability is generally

smaller than the typical resistance increase during evolution, which I don't think is that surprising. Instead, the more meaningful comparison to me is how trajectories differ within versus across genetic backgrounds. A shorter x-axis might help make this clearer. Additionally, providing a more intuitive number would make the result more tangible. For example, stating that "IC50 trajectories are on average X-fold more similar within backgrounds than across backgrounds". Getting an intuitive number like that was the intent of my original comment.

Like I said, while I encourage the authors to address these final comments, I do not think any of them warrant an additional round of review from my side. I therefore congratulate the authors on this work and look forward to reading the final published version.

Reviewer #3

(Remarks to the Author)
The Reviewers have addressed all my concerns, the manuscript reads extremely well and the science in it is fantastic.

Open Access This Peer Review File is licensed under a Creative Commons Attribution 4.0 International License, which permits use, sharing, adaptation, distribution and reproduction in any medium or format, as long as you give appropriate credit to the original author(s) and the source, provide a link to the Creative Commons license, and indicate if changes were made.
In cases where reviewers are anonymous, credit should be given to 'Anonymous Referee' and the source.
The images or other third party material in this Peer Review File are included in the article's Creative Commons license, unless indicated otherwise in a credit line to the material. If material is not included in the article's Creative Commons license and your intended use is not permitted by statutory regulation or exceeds the permitted use, you will need to obtain permission directly from the copyright holder.
To view a copy of this license, visit https://creativecommons.org/licenses/by/4.0/

POINT-BY-POINT RESPONSE TO REVIEWER COMMENTS

Reviewer #1 (Remarks to the Author):

Review on Petrungaro et al. entitled “Function-specific epistasis shapes evolutionary trajectories towards antibiotic resistance”

Whether global or idiosyncratic epistasis exhibit a stronger effect on the evolutionary trajectory of antibiotic resistance evolution is still unexplored apart from a couple of limited scale research with small sample sizes. Petrungaro et al. take on this challenge by implementing an automated high-throughput morbidostat platform capable of maintaining 864 parallel evolving populations simultaneously. This impressive experimental framework allows the authors to systematically examine how diverse genetic backgrounds influence both phenotypic resistance gains and mutational pathways under sustained selection.

The authors find that neither global epistasis nor idiosyncratic epistasis alone can fully account for the observed resistance trajectories. While a predominant part of evolution is highly repeatable, both at the phenotypic and the genotypic level and across widely different E. coli strains, they also identify clear cases in which perturbation of specific cellular subsystems constrains evolution into alternative, often less efficient, mutational trajectories. This “function-specific epistasis” is then explored in depth, and the manuscript provides several mechanistically compelling examples where disruption of efflux pumps, protein quality control, membrane biogenesis, or cell-shape maintenance reshapes the evolutionary landscape.

Overall, Petrungaro et al. present a carefully designed and thoroughly executed study that reveals how initial genotype, fitness, and resistance levels contribute to general trends in resistance evolution, while also uncovering the critical role of specific cellular functions in constraining or redirecting evolutionary paths. These findings substantially advance our understanding of antibiotic resistance evolution and will stimulate further research in both fundamental evolution and therapeutic intervention.

We thank the reviewer for their positive feedback on our work and their constructive suggestions for improvement.

Suggestions for Improvement:

Throughout the text, it may be misleading that on the one hand the authors emphasize that “overall resistance evolution is highly repeatable” across genetically distinct E. coli strains, while in other parts they state that “genetic background strongly influences resistance evolution.” It would be helpful to rephrase these points to distinguish clearly between: a general, global trend of high repeatability, and specific, function-linked deviations affecting a minority of genetic backgrounds. Explicitly emphasizing this distinction in the Abstract and Results would improve clarity.

We thank the reviewer for pointing this out. We have rephrased several sentences in the abstract (P1, L15-19) and several sections of the main text (P4, L11; P6, L28-29; P7, L21-23; P8, L13-14, 26-28; P11, L2-5) to clarify this distinction.

Several gene deletions that strongly alter resistance evolution (e.g., tolC, lon, dnaK, rodZ) have broad cellular roles, and their effects may involve general physiological constraints rather than epistasis with specific resistance mutations alone. Even though ancestral growth defects were minimized, some deletions may limit metabolic flexibility or stress tolerance, thereby reducing access to certain mutational paths or imposing costs on alternative routes. A brief discussion acknowledging these potential fitness trade-offs would help clarify whether slowed evolution reflects direct genetic

interactions or more global limitations on adaptability. This is especially relevant for “anti-evolution drug” implications.

The reviewer raises an interesting point. While the absence of growth defects ensures that the observed effects of gene deletions on evolvability are non-trivial, we agree that certain gene deletions may have global effects on bacteria, reducing their stress tolerance or metabolic flexibility, among other factors that could be important under antibiotic stress. We now address this point in the Discussion (P13, L4-11).

For nitrofurantoin, several populations approach the maximum soluble concentration during evolution. As this ceiling artificially compresses late-stage variation, the authors could explicitly mention how this affects the interpretation of the relatively deterministic NIT trajectories.

This is a good suggestion. Importantly, the conclusion that the NIT trajectories are relatively deterministic remains unchanged when all time points at which some populations reach the solubility limit are excluded. We have added a brief note explaining this (P4, L29-30). Following comment 2 from reviewer #2, we have introduced a new measure of phenotypic variability based on the pairwise separations between IC_{50} trajectories (Fig. S4, Methods P19, L1-38). We have performed the same analysis and statistical tests on the pairwise separation calculated over the truncated trajectories, excluding all time points that reach saturation. The significance of all tests remains unchanged.

Given the scale of the dataset and the complexity of the statistical analyses, making the raw IC_{50} trajectories and analysis scripts easily accessible (GitHub) would further enhance reproducibility and transparency.

As soon as the paper is published, we will make these trajectories accessible, along with a user-friendly explorer interface for navigating the data.

The selection procedure for the 258 KEIO deletions is detailed and methodologically sound, but only presented in the Methods. A short summary in the Results, highlighting how this set was chosen, would help readers understand the rationale behind the experimental design.

We have now added a brief description of the gene-deletion selection procedure to the Results section (P3, 37-40).

Figures 3 and S9 present rich mutational data. The authors might consider adding a brief textual summary grouping recurrent hits into functional categories (efflux regulation, target modification, LPS biosynthesis, oxidative stress), to make it more understandable for non-specialist readers.

Thanks for this suggestion. As suggested, we have now added a textual summary grouping recurrent hits into functional categories in Figs. 3 and S9.

On Figure 1A, the phrase “258 gene deletions, each 3 populations” is somewhat misleading. Using the same formulation as in the figure legend “258 gene-deletion strains in triplicate” would be clearer and more consistent.

We agree and have updated Figure 1A accordingly.

A brief mention in the main text of the mutator-screening procedure and its outcome (number of samples excluded) described in the Methods would help readers understand how mutators were handled.

We have added a brief explanation of the essence of the mutator-screening procedure and the number of excluded samples (P6, L32-36).

Even though ΔlacA is a straightforward reference strain, stating the rationale behind in the main text the same way as it is included in the methods, would make the experimental design more accessible to a general audience.

Following the reviewer's suggestion, we have included a sentence about the selection of *ΔlacA* in the Results section (P3, L40-43) .

Reviewer #2 (Remarks to the Author):

In this work, Petrungaro and colleagues evolve hundreds of single-gene knockouts from the Keio collection under selection by three different antibiotics. In each antibiotic, they observe that most knockouts follow broadly similar resistance trajectories, but a subset consistently deviates: these strains show a markedly reduced ability to evolve high-level resistance and thus act as evolvability suppressors. Through genomic analysis, they identify a small set of knockouts (notably tolC, dnaK, recA) that behave as robust evolvability suppressors across all three antibiotic regimes. They further demonstrate that small-molecule inhibitors targeting some of these common suppressors (tolC, dnaK) can modestly slow resistance evolution. More strikingly, they find a strong synergistic effect when domperidone is tested in knockouts of either subunit of the major efflux pump AcrAB, suggesting that analogous combinations of inhibitors might meaningfully constrain evolutionary escape routes.

Besides the general story of identifying evolvability suppressors and their potential clinical applications, the study offers at least three other interesting insights: 1) Experiments with E. coli UTI clinical isolates mirror the main patterns, which is a very wellcome result as reinforces the idea that laboratory evolution in model strains can generate principles relevant to pathogenic relatives. 2) Endpoint resistance level is highly idiosyncratic to the identity of the starting knockout, and only weakly related to its initial resistance level (in other words, they report a weak role for global epistasis). 3) Negative epistasis appears not the be the common explanation for the evolvability supressors: strong incompatibility between knockout backgrounds and typical resistance mutations is clearly supported for tolC but not for the other deletions tested.

We thank the reviewer for their careful evaluation and appreciation of our results, as well as for their detailed suggestions for improvement.

I like the authors' methods and analyses, and I find the main takeaways genuinely relevant. However, I have several concerns about some of the claims and, more generally, about how certain parts of the text and figures are presented. In addition, the manuscript could have benefited from more engagement with prior literature in the field. These issues reduce readability and hinder a full appreciation of the significance of the work. Here are my concerns:

1) In the introduction, I missed a clearer account aimed at a general reader that the quest for suppressors of antibiotic resistance evolution by single-gene deletion is an active research topic. This line of work was pioneered by their own previous study (Lukačišinová 2020 Nat Comm; and not just "Recent work" as humbly stated in line 10 of page 2), but also by Horinouchi 2020 Sci Rep and Gifford 2018 Nat Ecol

Evol. Although the authors cite these references, they do not clearly explain to a naïve reader how the present work inserts within and in what ways it advances the field.

We thank the reviewer for pointing this out. We agree that uninitiated readers need more information about previous work to understand our study's role in the field. We have revised the relevant sections of the Introduction and added several sentences to provide more context (P2, L22-44).

2) The second claim in the Results is that phenotypic resistance trajectories are highly repeatable. While I agree that the observed resistance trajectories are characteristic for each antibiotic, I find it problematic to reconcile with their claim that the y-axis in Fig. 2b and 2c is logarithmic and that, for example at time 200, the range encompassing 90% of the data spans almost two orders of magnitude (can we can this "highly" repeatable?). Overall, I miss a more precise metric of repeatability. For instance: what is the average distance between two trajectories sampled at random in resistance units (Euclidian distance, to keep it simple)? How much smaller are these distances within a strain relative to across strains? And how similar are these distances in E. coli UTIs compared with the KO strains? Clear, intuitive numbers would make this section more compelling.

The reviewer makes a very good point. It is true that the variability in resistance trajectories can exceed an order of magnitude. However, this variability is relatively small compared to the total increase in resistance over the course of the evolution experiment. To illustrate this, we followed the suggestion of the reviewer and introduced a new variability measure δ, which is the average absolute distance between two curves in logarithmic space, i.e., the average separation in orders of magnitude Note that we do not do this in linear space to avoid bigger absolute distances towards the end of the experiment dominating the measure. We chose to call the new variability measure δ "separation" instead of "distance" to avoid confusion with the Kolmogorov-Smirnov statistic used in the statistical tests we performed on their distributions.

In Fig. S4, we show these separations within and between various groups of strains (including those suggested by the reviewer) as well as the total increase over the evolution experiment for comparison. Briefly, we found that, for the knockout strains, separations within strains are significantly smaller than those across strains, indicating that single gene deletions can alter the evolutionary trajectories more than would be expected from the stochasticity of evolution alone (P4, L27, 29; P4, L46-P5, L1-3). The separations within knockout strains are larger than those within UTI strains for NIT, while there is no significant difference for MEC and TMP (P5, L13-16). However, we have only evolved a handful of UTI strains compared to the hundreds of gene-deletion strains. For all distributions, almost all values are far below the fold increase over the total duration of the experiment, showing that the phenotypic variability is generally much smaller than the typical resistance increase during evolution.

3) I really like Fig. 2a. It makes sense that the KO dataset is more variable and tends toward slightly lower evolved resistance, as these strains have fewer genetic options to "tinker" with. However, I missed an analogous plot for Fig. S12 (UTI strains). It's very interesting that these strains behave closer to the control lacA, perhaps because most polymorphisms they carry have been passed through the filter of natural selection in the wild, and are not as disruptive as some of the KOs. I would strongly suggest including the analagous plot for UTIs in the main figure, as this is an important takeaway from their study.

We have added the final fold-increase values for the UTI strains in Fig. 2a. Please note that there are far fewer evolution experiments for the UTI strains than for the gene-deletion strains. This makes it difficult to accurately estimate the distribution of fold increases. For this reason, we have chosen to plot the values rather than an analogous violin plot. The values are now shown as black stars over the original distributions for the reference and the gene-deletion strains. As mentioned in our response to point 2, we have further investigated how close the evolutionary trajectories of UTI strains are from those of the

control *ΔlacA* in Fig. S4d-f (which replaces the previous Fig. S12). This analysis suggests that lab strains could serve as valuable proxies for clinical isolates in the study of evolutionary dynamics (P5, L13-16; P13, L16).

4) Page 10, lines 4–23. The authors argue that, unlike in many microbial evolution studies, global epistasis explains only a small fraction of genotype effects on resistance gains. I missed a clearer account of the relevant literature here, as this remains an active and open question: on the one hand, Card 2019 PLoS Biol, Horinouchi 2020 Sci Rep, and Jangir 2022 eLife (PMID: 35943060) do not observe global epistasis, whereas Lukačišinová 2020 Nat Comm and Sánchez-Maroto 2025 PLoS Genet (PMID: 40853985) do. We don't really know how to reconcile these differences. The authors gloss over this rich and recent literature with the broad statement that diminishing-returns epistasis is "typically not observable" in short antibiotic-resistance evolution experiments (Card, Horinouchi). But Horinouchi 2020 spans up to 192 hours, which is comparable to the current study and also to Lukačišinová 2020! In addition, Sánchez-Maroto 2025 is a two-step adaptive walk similar to Card 2019, yet they see the pattern while Card and cols do not. Please revise.

We agree with the reviewer that this point requires further discussion. Our statement about short evolution experiments primarily applies to the Card (2019) paper. Most of the data shown in Horinouchi (2020) were collected over a period of up to 5 days (120 hours). However, the number of generations the cultures undergo is considerably smaller than in our experiments over the same time, since the passages in the experiments in Horinouchi (2020) are performed every 24 hours, letting the bacteria remain in stationary phase for a period of time. In part due to this, resistance typically increased by less than 10-fold in about 120 hours, which is considerably less than in our dataset. Therefore, resistance is probably far from saturating, which likely makes diminishing returns epistasis harder to detect.

We have revised and expanded the relevant part of the Discussion (P11, L7-38) to clarify this point and to better explain the main limitations of previous experiments that could be related to the absence of global patterns in those studies. In the revised version of our manuscript, we now provide a more thorough review of the previous literature on this topic, and we have included the additional articles mentioned by the reviewer.

5) Beyond including a more accurate description of the debate described above, the authors are missing here a major opportunity. Kryazhimskiy 2014 Science famously shows that global epistasis diminishes with time. Given the richness of the authors dataset, I wonder what would happen if the authors reproduced Fig. S3 for shorter timescales, say 50, 100, 200, 400 hours. This analysis is straightforward. In addition, Sánchez-Maroto 2025 PLoS Genet reports that the global trend becomes much clearer after removing evolvability-suppressor lineages. What would happen here if the authors removed lineages that strongly deviate from the common trend? If global epistasis still failed to appear in earlier time points without evolvavily supressors, that would be a valuable result. As stated, this is a missed opportunity to make a deeper contribution at a fundamental level, and I encourage the authors to explore it.

We thank the reviewer for pointing out the opportunity to explore this interesting point further. We have now included two new panels to Fig. S5 (previously Fig. S3), in which we show the data after removing the evolvability suppressors. While we observe that the trend indeed becomes slightly stronger, the correlation coefficients remain low. In any case, these correlations are much weaker than those observed in the other studies that detected a clear global epistasis trend. Regarding the pattern for shorter timescales, we have explored this opportunity and summarized the results in the figure below. Since we did not observe a clear trend in the change of correlations over time, we would prefer not to include this analysis in our manuscript. However, we are open to including it if the reviewer finds it informative.

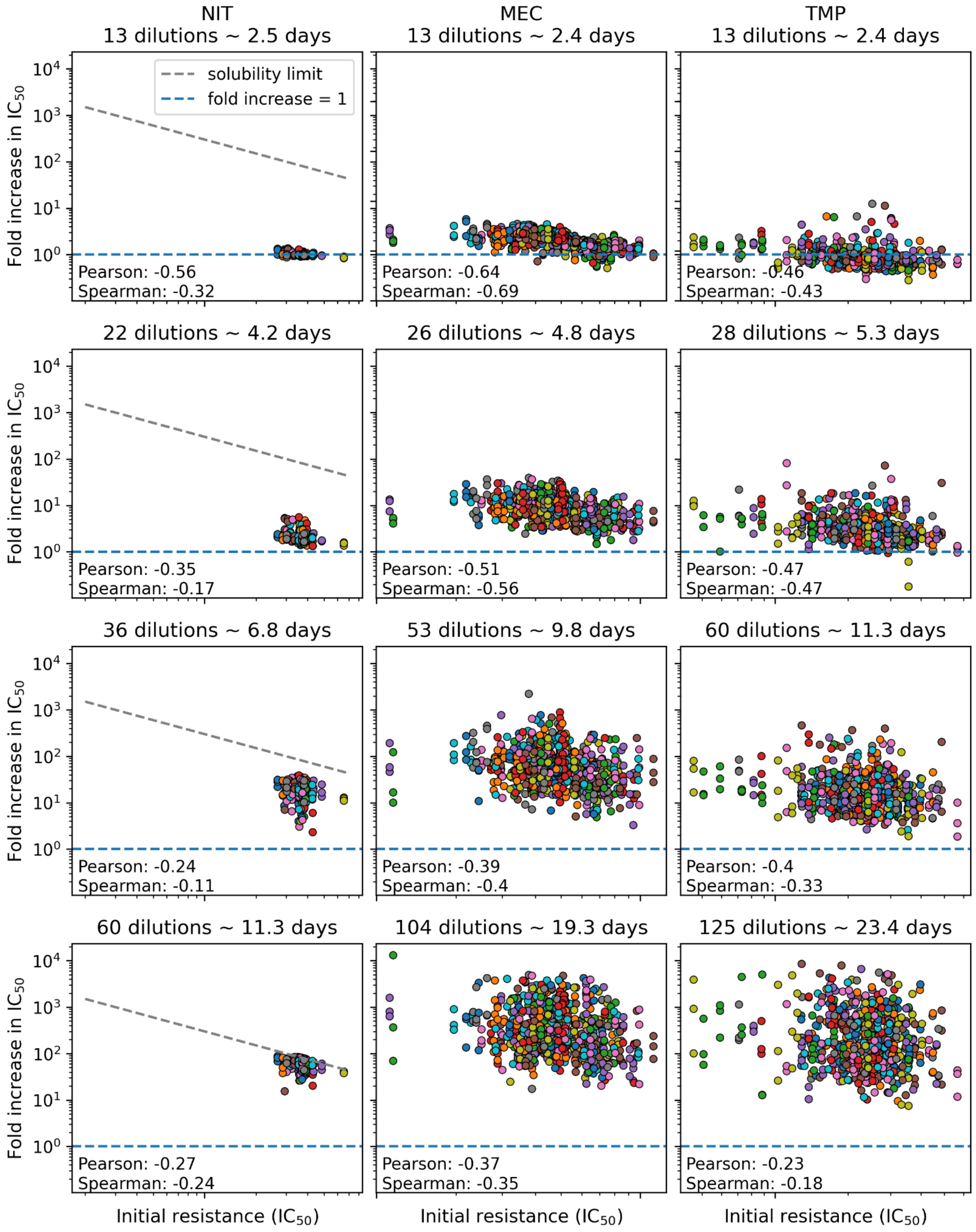

Fig. R1: Fold increase in IC_{50} versus initial resistance for different time points. Time points were chosen according to the duration of the experiments; the last row corresponds to the last row in Fig. S5. Significant strains as of Fig. 4 and mutators are excluded. IC_{50}s shown are the average of 5 dilutions to smooth fluctuations (title dilutions ±2 dilutions).

6) Fig. 1e–g is difficult to interpret at first glance because the x- and y-axes do not share common ranges or scales. Using consistent ranges would immediately illustrate that patterns differ across treatments in both speed and magnitude of fitness gains. I also believe it would be useful to show explicitly that the NIT experiment was cut short due to solubility limits, this helps readers understand the experimental

constraints (no shame in this!). Finally, please use more human readable units such as days instead of hours.

We have updated Fig. 1 according to the reviewer's suggestions, and we have changed the units from hours to days throughout the manuscript.

7) Page 5, lines 12–15: "Consistent with recent observations in strains from the E. coli Long-Term Evolution Experiment (19,42), the effects of genetic background on the evolvability of antibiotic resistance appear to be mostly idiosyncratic...". As noted in point 4, the authors should more broadly discuss the literature on idiosyncratic versus predictable patterns, beyond the two LTEE papers.

Please, see also our answer to point 4. We have expanded our discussion of idiosyncratic versus predictable patterns, taking into account the literature beyond the two LTEE papers in the Discussion (P11, L7-38). We prefer to avoid a longer discussion of this topic in the Results section because it would disrupt the flow. However, we have revised the sentence the reviewer pointed out so that it briefly mentions contrasting results from other relevant studies (P5, L32-33).

8) Page 6, line 48: "Deletions of components and regulators of multidrug efflux pumps ... drastically reduced resistance evolution for all three antibiotics tested here (Fig. 4; Fig. S4–S6), as well as for tetracycline and chloramphenicol (Lukačišinová)". I missed here mention and discussion of Horinouchi 2020 Sci Rep, which also identified robust evolvability suppressors across three antibiotics (cefixime, ciprofloxacin, chloramphenicol), though the genes differ (lysR, arcA, gutM, fur). If future work across multiple antibiotic classes is conducted, do the authors expect general rules for evolvability suppressors? Perhaps not universally, but possibly for antibiotics sharing resistance mechanisms or physiological targets?

One reason the two studies identified different genes is that they tested different selections of gene deletions. Horinouchi (2020) focused on transcription factors, whereas we covered diverse cellular functions (Fig. S1). Apart from the different antibiotics used, there are technical differences in how the evolution experiments were performed, which may lead to different outcomes. Notably, some of the gene deletion strains (e.g., *arcA*) identified in Horinouchi (2020) exhibit severe growth defects and would have been excluded from further investigation in our study.

As suggested by the reviewer, we now mention Horinouchi (2020) in the corresponding lines (P7, L37-40) and we have included additional discussion of this work and the aforementioned points in the Discussion section (P12, L25-30). As we wrote there, yes, we expect gene deletions that disrupt resistance mechanisms shared by several antibiotics to act as evolvability suppressors generally for those antibiotics. One example is the gene deletions of efflux pump components and their regulators (*acrB*, *tolC*). However, we believe that for *tolC* this might be even more general, as efflux pumps are not the main resistance mechanism for TMP and NIT for which this deletion arose as an evolvability suppressor. Further, we expect that chaperones, such as *dnaK* (and probably also some proteases) may suppress evolvability more generally for many different antibiotics.

9) Page 8, lines 17–36: This passage is difficult to follow; at times it comes across as poorly written, with long sentences containing multiple clauses and ambiguous subjects. For example, the sentence beginning "we hypothesized that deleting the specific genes from evolved strains carrying those resistance mutations would reduce their resistance to levels similar to those of populations that evolved from ancestors with the specific gene deletion". It also contributes to the confusion that it seems that when the authors refer to "Fig. 3b; Fig. S9" in line 24, they actually mean Fig. 5 and Fig. S10. Even if so, the genes mentioned do not fully match what is shown in Fig. 5g and Fig. S10 (where is yobH?). Please revise and try to use shorter sentences.

We thank the reviewer for pointing out the misreferenced figures and apologize for the error. We have corrected them. Indeed, we meant Fig. 5g,h and S10 (now S12). As the reviewer suggested, we have also substantially revised this paragraph to make it more accessible. In particular, we have rephrased it using shorter sentences where possible (P9, L11-24). We have included *yobH* in Fig. 5g. In line with minor point 15, we have also revised the x-axis tick labels in Fig. 5h and its legend to better clarify the points explained in the sentence mentioned by the reviewer.

10) Related to the work presented in Fig. 5g and 5h: the text should specify the genotype of the evolved reference strains from which those genes were deleted. With 36 replicates available, what guided the selection of these particular 3 lineages? The Methods describe how a clone was chosen for the selected ΔlacA evolved populations (the most typical dose–response curve), but nothing is said about the choice of these 3 lineages in the first place. It would be very interesting to know the resistance mutations these selected ΔlacA lineages accumulated. Had the authors chosen others, maybe lon and yobH KOs would have had more effect in the evolved lines?

We selected these three populations to cover different resistance levels within the 36 evolved ΔlacA populations. Specifically, we selected one population with the highest resistance, one with intermediate resistance, and one with the lowest resistance. In the revised version of the manuscript, we have included a description of this selection process in the Methods section (P18, L8-10). Following this and the next suggestion from the reviewer, we have included these evolved *ΔlacA* populations in Fig. 5d–f, which show their mutated genes. More information about the mutations these populations have accumulated beyond the gene level can be found in Table S1. They can be identified by their symbols in the "symbol in Fig. 5 and S12" column. We have now also explicitly specified this in the Methods section (P18, L10-12).

11) Fig. 5d–f is not very clear. Why are there blank spaces in the heatmap? What do the "..." separators represent? If the point is to show that replicates from low-evolvability backgrounds tend to share uncommon mutations, why not illustrate one example (e.g., pepN), and compare its mutation profile with several ΔlacA endpoint isolates and with endpoint isolates from non-suppressor backgrounds that harbor the most repeatedly hit mutations?

The blank spaces in the heat maps in Fig. 5d–f indicate that the respective loci were not mutated. The "..." separators indicated that many genes were omitted from the rank-ordered list of genes on the x-axis, which is ordered by how frequently they were mutated. We thank the reviewer for their suggestion of showing several *ΔlacA* endpoint isolates alongside one example from the low-evolvability backgrounds. Following this suggestion, we now show the mutation profile for the same three evolved *ΔlacA* replicate populations used in Fig. 5h. We believe this improves the clarity of the figure's message. However, adding more non-suppressor populations would make the figure too crowded. Similarly, we have now removed the "..." together with the rightmost panels in Fig. 5d–f, which showed the higher-ranked mutations before, for consistency with Fig. 3a and Fig. S8. We rather refer the reader to the complete mutational data provided in Table S1 for a more detailed comparison of mutations across specific endpoint populations.

12) Page 8, line 45: "Further elucidating the underlying molecular mechanisms can be difficult." I am not fully convinced by this. I will not insist, as the study already contains an enormous experimental effort... but testing the most common mutations specific to suppressor backgrounds (and absent from the general pool) in both the suppressor background and a ΔlacA background could reveal cases of positive epistasis (i.e., mutations only beneficial in the presence of the evolvability supressor KO).

We agree that testing the mutations specific to suppressor backgrounds in both the suppressor and the reference could reveal relevant epistatic interactions. We appreciate the reviewer's understanding that this would require additional experimental efforts beyond the extensive dataset already collected and

would go beyond the scope of this manuscript. However, it is certainly an interesting avenue for future work. We have added a sentence about this point to the Discussion section (P13, L9-11).

Minor points

13) Page 6, regarding WGS: "For each antibiotic, the selection included 5 to 8 replicates of the reference strain and all three evolutionary replicates for each of at least 30 gene-deletion strains whose resistance trajectories clearly diverged from the reference strain". Selecting strains that "clearly diverged" biases the sample toward less repeatability. I think this should be highlighted, as it makes the resulting parallel evolution all the more remarkable.

We agree with the reviewer. We have added a sentence to highlight this point (P6, L28-29).

14) Page 8, line 3: "Notably, for NIT, disruption of the Lon protease consistently prevented...". I think it would be neat to mention that loss-of-function mutations in lon were previously proposed to impose a hard limit on evolvability in a psychrophile adapting to elevated temperature (Toll-Riera 2022 Sci Adv; PMID: 35857489).

Thank you for pointing out this interesting finding. We now mention this similarity to previous results and cite the Toll-Riera paper (P8, L42-44).

15) I really like the experiments shown in Fig. 5g and 5h, testing how deleting an evolvability suppressor affects resistance levels in evolved strains. However, I think the caption can more clearly explain that Fig. 5h compares: (i) how much lineages starting with a suppressor mutation achieve lower resistance during evolution, versus (ii) how much resistance drops when deleting these same genes from a ΔlacA-evolved population. It is a neat and insightful comparison but undersold, in my opinion.

We agree that this can be made clearer. We have revised the caption of Fig. 5 accordingly, and explicitly incorporated the distinction between (i) and (ii), which is now mentioned in the x-axis tick labels in Fig. 5h.

16) Fig. 3: "Numbers and color intensity indicate the number and percentage of replicate populations with the mutation, respectively." I do not see a legend for the percentages. Please include one.

We have revised Fig. 3 to include a legend indicating the percentage of replicate populations.

Reviewer #3 (Remarks to the Author):

The authors present a compelling manuscript that addresses the important question of how epistatic interactions influence the evolution of antibiotic resistance. They perform high-throughput experiments to track resistance evolution over time for three different antibiotics, using hundreds of gene-knockout strains. They used this data to estimate the extent to which variation in resistance evolution is determined by genotype, a key finding. Furthermore, by sequencing a subset of the final evolved populations, they add substantial evidence and novel detail into how gene deletions influence a strain's potential mutational space for antibiotic resistance. The experiments are explained clearly, the main text figures are well-made and intuitive to understand, and the results are compelling. However, in certain places I believe the findings do not fully support the authors ultimate conclusions. Additionally, a strange pattern of extreme variation is present in a subset of the supplementary data which is not

adequately explained. If these problems are addressed, it would improve an already strong piece of work.

We thank the reviewer for carefully inspecting our manuscript and providing detailed comments. We believe these comments have helped us improve our work.

Major comments:

1. Sometimes I think complicated and potentially contradictory results are simplified too much in order to create a clearer or more impactful narrative. This is most obvious in the discussion.

We have carefully revised the wording in our manuscript and have now rephrased several sentences to address all of the reviewer's comments. Please see our responses to each of the items a-d below (P3, L2, L10-12; P6, L46; P7, L2, L21, L45-46; P8, L1, L12-15, L17, L28, L36, L39; P9, L18; P10, L43; P11, L1-5, L43-44; P13, L16; P37, L2).

a. P9, L41-45. I think the authors should be careful saying they identified specific functions that influence evolution, as they don't do any kind of functional enrichment analysis. It's probably fine for the "across antibiotics" functions, as they have multiple genes for each and the functions are all known to influence resistance for various antibiotics. But the second sentence saying certain functions only influence resistance for specific antibiotics feels like too big a claim for the data. In the results, only one gene each is mentioned for those functions, and the functions are not labelled in Fig. 4a.

We agree that our statement that certain functions only influence resistance for specific antibiotics did not clearly convey our intended point. We have rephrased the sentence in question to soften this claim and provide a more precise statement in the Discussion section (P10, L43-P11, L2). We have also labeled the relevant functions in Fig. 4a. While it is true that, in some cases, only one gene deletion strain meets our stringent criteria for detecting effects on resistance evolution, additional genes pointing to the same function with weaker effects on resistance evolution corroborate our statement for all functions mentioned in the Results section. For example, not only *ΔseqA*, but also *Δhda* and *Δdam*, support the view that the regulation of chromosome-replication initiation affects NIT resistance evolution (Fig. S11, previously Fig. S4). Similarly, not only *ΔdnaK*, but also *ΔdnaJ* and *Δtig*, support the view that chaperones (protein folding) affect NIT resistance evolution (Fig. S11, previously Fig. S4). Similar examples exist for most other functions that we mention for the other two antibiotics (Fig. S9, S10, previously Fig. S5, S6). We have added additional example genes to the relevant paragraph in the Results section (P7, L45-P8, L1) to clarify this point.

b. P10, L1-2. Did the small molecule inhibitors partially recapitulate these effects? The authors didn't sequence the populations evolved in presence of inhibitors, so they don't know if it influenced resistance evolution on a genotypic level. And neither of the inhibitors (for any of the antibiotics) changed the final IC_{50} for the ΔlacA reference strain, which would be akin to recapitulating the effect of a gene deletion.

The reviewer is correct. We have now rephrased the relevant sentences (P11, L3-5; P3, L10-12) to make a more precise statement.

c. P10, L28-29. The authors don't adequately back up the statement that the populations converged on the same resistance mutations, as they only looked at whether the same genes were mutated. Looking at the dataset, 94% of mutations only occurred in one population, and 3% only in two populations. If resistance mutations in most of the top gene targets are highly disruptive mutations, it makes sense the authors wouldn't see the exact same mutations. This should be acknowledged and more careful word

choice is needed to make clear the authors only look at the genotypic repeatability of evolution at an individual gene level, not an individual mutation level.

We thank the reviewer for pointing out this issue. We have rephrased this explanation stating that we analyze mutations at the gene level, where we find considerable convergence (P11, L43-44). To avoid confusion, we have changed the wording in various places throughout the manuscript to be more precise (P6, L46; P7, L2; P8, L13-15,17, 28, 36, 39; P13, L16). We have also rephrased the title of the inset in Fig. 3a.

d. Following the above point, the authors also sometimes use the term "mutational trajectories", which slightly misrepresents the fact that they only have sequencing data for the final populations, not over time. This is particularly confusing because they use the term "resistance trajectories" to accurately describe the longitudinal data for IC50s.

We agree that using the term "mutational trajectories" might create the impression that we have sequencing data over time. To avoid this, we revised the wording in several places (P3, L2, 10; P7, L21; P8, L12, 14; P9, L18; P11, L3; P37, L2). We now consistently talk about mutational "paths", instead of using "trajectories", the latter of which is used only for our time-resolved IC_{50} data. We believe that this wording resolves the confusion while emphasizing that, although we only have sequencing data for the final populations, the different mutated genes in the final populations still reflect different genetic paths toward resistance.

2. What is causing the dramatic drops and spikes in the IC50 measurements over time, seen in the resistance trajectories (most prominently in Fig. S6 for TMP but also Fig. S5 for MEC)? The only mention of it is P7, L24-25 saying there's high variability between replicates (not within replicates over time). Steps are taken to not show this in the main text figures by applying smoothing functions (methods: P18, L1-14), where these drops and spikes are described as "noise in the data". But these are consistent variations of orders of magnitude. Why does this happen? Why does it happen more with specific antibiotics? The lack of explicit acknowledgement and clearer explanations raises questions about the validity of the underlying data.

The dramatic drops and spikes in the IC_{50} time series are due to a technical limitation of our experiments. We run 864 independent experiments simultaneously in our robotic platform. The target antibiotic concentration in each experiment's well is mixed from two reservoirs, one containing only media and the other containing media at one of two available antibiotic concentrations, as explained in the Methods section (P16, L20-24). This technical limitation stems from the fact that, although we update the concentrations in the antibiotic reservoirs to best fit the needs of all experiments, the dynamic range of concentrations that can be prepared from the two reservoirs is limited by the range of volumes that the liquid handling robot can pipet. Initially, this constrained range of concentrations can cover all IC_{50}s of the evolving populations. Over time, however, many fast-evolving strains reach high IC_{50}s, while some slowly evolving strains lag orders of magnitude behind. The difference in IC_{50} between the slow- and fast-evolving populations exceeds the range that can be covered with our two antibiotic reservoirs. In these cases, the slow-evolving strains were first diluted using medium without any antibiotic and then set to the lowest feasible concentration, which is usually still higher than their IC_{50}. This procedure causes the sudden drops (observed when diluting without antibiotic) and spikes (observed when setting the concentration to the lowest feasible one) in some of the low-resistance trajectories. Importantly, this technical limitation does not prevent the reliable detection of slow-evolving strains – it only limits the quantitative estimation of their resistance evolution rate later in the experiment.

This technical limitation of our experiments affects some antibiotics more than others. It leads to more pronounced variations for antibiotics with large differences in IC_{50} between fast- and slow-evolving strains because a wider dynamic range of drug concentrations must be covered. For NIT, solubility

limits the maximum drug concentration. Consequently, the dynamic range that our reservoirs need to cover is relatively small, and the NIT evolution experiments are less affected by this limitation.

We have now explicitly acknowledged this technical limitation by including a similar explanation in the Methods section (P16, L25-37).

3. Throughout the manuscript, but particularly in the introduction, more clarity and consistency is needed when discussing the different types of epistasis. A variety of terms - global epistasis, microscopic epistasis, diminishing-returns epistasis, idiosyncratic epistasis, and function-specific epistasis - are used at various points. “Global” and “diminishing-returns” seem to be used interchangeably, despite not being exactly the same. Is “function-specific” simply a subset of “idiosyncratic”? These terms need clearer definitions and if using fewer distinct terms is possible it should be preferred.

We agree that the different types of epistasis may confuse some readers. Throughout the manuscript, we now consistently use the term “global epistasis” and only mention “diminishing-returns” when referring to this specific type of global epistasis, mostly in the Discussion (P1, L34-35; P2, L13; P2, L18-19; P5, L22; P10, L39, P11, L7-38). We also removed the term “microscopic epistasis” from the introduction and instead rephrased the relevant sentence, following the reviewer’s suggestion to minimize the use of technical terms. In the same spirit of avoiding unnecessary terms, we have also removed the word “contingency” throughout the manuscript.

We would not describe function-specific epistasis as merely a subset of idiosyncratic epistasis. The key difference is that function-specific epistasis enables predictions, albeit partial, of resistance evolution based on the functions of perturbed genes, whereas idiosyncratic epistasis, by definition, has unpredictable effects on evolution. We have added a new supplemental figure (Fig. S1), which illustrates the key differences between the relevant types of epistasis (mentioned in P1, L34; P2, L1; P3, L9; P7, L41).

4. It’s stated in the methods that all the experiments were performed at 30°C rather than optimal temperature of 37°C for E. coli. Why was this choice made? It should be stated in the main text and justified in the methods. It does not make the results any less valuable, but more discussion of the potential consequences is needed – particularly given that clinical UTI strains are used, and that comparisons are frequently made to results from published studies that used 37°C. Some caveating of those comparisons should be included, as, for example, genes are differentially expressed between the two temperatures.

It is good to clarify this point. We primarily performed the experiments at 30°C for technical reasons. The robotic system used for the evolution experiment is in a climate-controlled room at 30°C. Further raising the temperature in this room is not feasible because the instruments that make up the integrated system and their control computers are not approved for higher temperatures. Although we could set the temperature inside the incubator, where the microtiter plates with bacterial cultures are located most of the time, to 37°C, we decided against it for several reasons. First, doing so would cause temperature fluctuations whenever the plates are unloaded from the incubator. This can subsequently cause condensation on the plates when they return to the warmer incubator from the colder room because the incubator is kept at high humidity (>95%) to prevent evaporation. Such condensation would be a severe problem for the optical density measurements. Second, 37°C would result in faster growth, requiring more frequent dilutions to maintain exponential growth of the bacterial populations. This would inevitably increase the robotic system’s error rate, making the experiment (even) more challenging. Note that numerous other studies using similar equipment have performed experiments at 30°C for essentially the same reasons (Toprak *et al.* Nat Gen, 2012; Hegreness *et al.* PNAS, 2008; Shachrai *et al.* Molecular Cell, 2010; Lukačišinová Nat Comm, 2020). Therefore, working at 30°C ensures that our results are comparable to those of previous studies (Lukačišinová Nat Comm, 2020). Importantly, 30°C is not a stressful condition for *E. coli*, and most aspects of its cell physiology are very similar to those

at 37°C (Bremer and Dennis, Ecosal 2008). However, we agree with the reviewer that comparisons to results from studies performed at 37°C could be affected by this.

We now state in the main text that the experiments were done at 30°C (P3, L24). We have further added a more detailed explanation of this choice, as well as the caveats it may introduce for arguments based on previous results obtained at 37°C, in the Methods section (P14, L1-10).

5. There are several places where it seems the wrong figure is cited, and many places where figures (particularly supplementary) are cited out of order (e.g. Fig. S12 cited before Fig. S3). I have pointed out several of these places in the minor comments, but the manuscript needs to be fully checked to ensure every figure citation is correct, and that figures are labelled in the order they appear in the main text.

We apologize for these errors and thank the reviewer for pointing them out. We carefully checked the manuscript to identify instances where the incorrect figure was cited. The following were corrected in the revised version of our manuscript:

- P4, L45: Fig. 2b → Fig. 2c
- P5, L42: Fig. 2i,j → Fig. 2h,j
- P5, L44: Fig. 2h → Fig. 2i
- P8, L41: Fig. 5c → Fig. 5d (previously Fig. 5f)
- P8, L45: Fig. 5b → Fig. 5e
- P12, 34: Fig. 5b,c → Fig. 5d,e
- P12, L36: Fig. 5e-g → Fig. 5g-h, Fig. S12 (previously Fig. S10)

We have also swapped Fig. 5a with Fig. 5c and Fig. 5d with Fig. 5f, to address minor comment 24g below.

All references to figures should now be correct. Additionally, we have rearranged the order of the supplementary figures so that they are labeled in the order in which they appear in the main text. In particular:

- Fig. S1 and Fig. S4 are new. Fig. S4 has replaced the previous analysis on Fig. S12.
- Fig. S2 remained Fig. S2
- the previous Fig. S3 → is now Fig. S5
- Fig. S4 → Fig. S11
- Fig. S5 → Fig. S9
- Fig. S6 → Fig. S10
- Fig. S7 → Fig. S6
- Fig. S8 → Fig. S7
- Fig. S9 → Fig. S8
- Fig. S10-S11 → Fig. S12-S13

6. Table S1 appears to be missing?

We are uncertain about this issue. Table S1 was included in the submitted files, and it appears that the reviewer consulted it for point 1c. In any case, we will make all relevant data publicly available and provide all relevant files.

Minor comments:

1. P4, L23-26. The reference vs knockout distributions do not look similar to me. And the next sentence points out that they are different, as resistance gains are lower in knockout strains. Statistical tests are

needed to determine if the difference between reference and knockout distributions is significant. If the distributions are significantly different, then the first sentence is hard to justify.

We agree with the reviewer that stating that the distributions of resistance gains are similar between the reference and knockout strains is not ideal. Accordingly, we have rephrased this sentence to be more specific on which are the differences and similarities between the distributions (P4, L39-41). For completeness, we have performed a Kolmogorov-Smirnov test, which revealed that the distributions are significantly different, except for TMP (NIT: $p<10^{-7}$, MEC: p-value=0.047, TMP: p=0.13). However, please note that we are not claiming that they are identical, only that they show some similarities, which we now specify.

2. P4, L39. Assuming the authors have sequenced the genomes of the seven isolates used for this experiment. They should report on the % of genes shared between the strains.

We did not sequence the genomes of the specific UTI isolates we used in this experiment. Therefore, the value of 75% of shared genes is a typical value that was reported in (Schreiber et al. Science Translational Medicine, 2017), as cited.

3. P4, L41-44. The resistance trajectories do not seem remarkably similar for NIT in Fig. 2f, it looks like the isolates consistently evolved resistance faster. This leaves me extra confused by the p-values mentioned in the text, which say NIT is the least significant. Why are S12 and significance tests only looking at resistance levels at the end of the experiment, which misses the earlier-occurring resistance? Could the low different between ΔlacA and UTI isolates at the end of the experiment for NIT be related to the solubility limit?

We agree with the reviewer that the UTIs consistently evolved slightly faster for NIT, at least initially. However, we realized that several UTI trajectories were missing from Fig. 2f, and the ones shown were not representative of the entire set of trajectories included in the analysis of Fig. S12 in the previous version of the manuscript. We have now added the missing trajectories to Fig. 2f. It is also correct that the solubility limit that is reached for NIT at the end of the experiment can artificially reduce the differences between the reference strain and the UTI isolates. To account for this and the mentioned initial differences, we performed a new analysis comparing the resistance trajectories over the entire time course of the experiment. This analysis is shown in the lower panels of Fig. S4, which replaces the previous Fig. S12. We have rephrased the relevant sentences to explain the results of this new analysis, which more clearly supports our previous statement on the similarity between the *ΔlacA* and UTI isolates' evolutionary trajectories (P5, L13-16). Importantly, all results remain unchanged when comparing the trajectories after removing all time points where the solubility limit was reached. This indicates that the similarity of the *ΔlacA* and UTI isolate trajectories is not due to the solubility limit.

4. P5, L1 Has this claim been shown or justified so far? No figure is cited. I guess the following figures explain it, but it's a strong claim to not provide immediate evidence.

Thanks for pointing this out. We added a reference to Figs. 2h-j and Fig. S5 to this sentence (P5, L21-22).

5. P5, L22. It's clear in the figure, but please make it clearer in the text that although initial resistance is a subset of initial genotype, it is not a very large subset. As is, talking about the high importance of initial genotype "...including initial resistance" feels contradictory to the previous paragraph about how initial resistance didn't matter much.

We have rephrased this sentence (P5, L40-41) to clarify the point raised by the reviewer.

6. P6, L42. Fig. 4a has 186 total strains in the Venn diagram, out of 258 strains total. So this does not seem to be "rare"?

The reviewer is correct that the instances mentioned in P6, L42 are not “rare” since we refer to all three antibiotics. This claim holds true for TMP and MEC, but not for NIT. We have rephrased the sentence changing “rare” for “several” (P7, L22).

7. P7, L2. Fig. 4 as a whole is referenced here, but Fig. 4b-d are never specifically referenced. This is probably fine, as Fig. 4b-d are clearly just showing the IC_{50} curves for a subset of the data. But because they are not mentioned specifically in the main text, I am left with some questions:

a. why pick these specific three gene deletions? I see each fits into one of the categories you mention in L3-L4. But LPS biosynthesis category (lpxM) is not shown.

b. Also why only showing us gene deletions that evolved resistance more slowly? Would also be helpful to see ones that consistently evolved more quickly (e.g. mutL, uvrD).

c. More information about why these specific deletions were selected should be added briefly to figure legend, and explicitly in methods.

We chose three example gene deletions from the middle circle because the effect is most clearly seen by looking at the IC_{50} curves. The effect of some of the other gene deletions might only become evident after performing the described statistical test. As the reviewer suggested, we included *ΔlpxM* in these plots in the revised Fig. 4 and added a brief statement in the caption of Fig. 4 explaining how these gene deletions were selected. We have additionally included *Δhfq*, to have one representative of each of the functions shared between all three antibiotics. The IC_{50} curves for all other gene deletions that appear in the Venn plot are shown in Fig. S9-S11 (previously Fig. S4-S6). Curves for some strains that evolved faster are also shown in Fig. S9-S11. We chose not to include these in Fig. 4 because most of them are either due to the deletion of a gene where a loss-of-function mutation occurs in the usual mutational path to resistance, or due to an increased mutation rate. The former phenomenon is entirely expected and the latter has been well studied and is not the focus of our study. Please note that the entire dataset (for all antibiotics and all gene deletion strains) will be openly available.

8. P7, L17. The authors give an example gene for every other functional category, but not response to DNA damage. Because Fig. 4a; Fig. S4 is referenced immediately after this text I looked for them there, but response to DNA damage is not mentioned in there either.

Thanks for pointing this out. We have revised the relevant sentence and added specific examples of genes involved in the response to DNA damage (SOS response): *uvrA*, *uvrC*, *holC*. These genes, which affect resistance evolution to NIT, are also shown in Fig. 4a or Fig. S9. The reviewer is correct that DNA damage is not explicitly mentioned in Fig. 4. However, it is part of the broader category labeled “Mutation rate altering”. We have also added the category symbols to the genes mentioned in Fig. 4.

9. P7, L24-25. The greater divergence between replicates isn't obvious to me from Fig. 1g, as the variability metric is comparable to MEC in Fig. 1e. And in Fig. S4-6 it’s hard to visually compare replicates. Although I do see the high pattern of high variation (see major comment) is very present in Fig. S6.

We have revisited this statement in light of the variability metric mentioned by the reviewer and also more carefully using the variability measure introduced in the revised version of our manuscript (Fig. S4). We agree with the reviewer that the divergence of resistance trajectories between replicates for

TMP is only clearly greater compared to NIT, but not compared to MEC. Accordingly, we have removed this sentence.

10. P7, L26-28. This feels like an insufficient explanation for TMP being unique, given that Fig. 3a shows that TMP has the highest mutation diversity and that NIT mutations are also mostly in two mechanism-specific genes (nsfA/B).

We also agree with this point. In these sentences, we speculate about why just one gene deletion clearly affects the resistance trajectories uniquely for TMP, but we do not have a definitive answer. Accordingly, we have revised this paragraph and have toned down this sentence (P8, L5-10). The reviewer is also correct that TMP has the highest diversity of genes mutated; however, *folA* mutations are still the most frequent by far (Fig. 3a; the fraction of samples with mutations in *folA* is much higher than that of the most mutated gene for MEC).

11. P7, L32-33. This is an interesting result, but there's no figure cited to back it up. Fig. 3a shows that the same genes were mutated in most samples, but this doesn't tell me anything specifically about the deletion strains that had slowed resistance evolution. Are the most-mutated-genes more or less common in slow-evolution strains compared to other strains?

The reviewer is correct that we only describe examples of gene-deletion strains that evolve slowly acquiring different mutations later in the text. This paragraph emphasizes that gene deletions significantly alter the mutations that fix during resistance evolution, as corroborated by the analysis described. Therefore, we rephrased this paragraph to clarify that point and moved all results that describe the mutation profile of specific gene deletions that slow resistance to the following paragraphs (P8, L13-46; P9, L1-6). In Fig. 5d-f, we show the genes mutated in evolved gene-deletion strains for one example of those evolving slower for each antibiotic. We compare them with the genes mutated in the reference strain and in most samples. These examples show substantially altered mutational paths. In the most extreme cases (Fig. 5d,e), none of the most mutated (i.e. multihit) genes are mutated.

a. Also, be careful with the wording of "...carry a subset of the common beneficial mutations". This may well be true, but so far the authors have only shown that the populations share common genes that are mutated. The authors haven't shown if the specific mutations within those genes are the same.

We have changed the wording (P8, L13-14). Please also see our response to major comment 1c above.

12. P8, L26. What are the common resistance mutations?

Here, we refer to “common resistance mutations” as mutations in genes that frequently occur in replicates of the reference strain and in most gene deletion strains, as shown in Fig. 3a. For clarification, we have rephrased the sentence and added a reference to Fig. 3a (P9, L15-16).

13. P8, L28. Fig. 5g only shows data for NIT, although previous sentence says they did this for all antibiotics. Can the authors include the same dose-response curve plots for TMP and MEC in the supplementary and cite here with Fig. 5g.

We have included analogous dose-response curve plots for TMP and MEC in new panels in Fig. S12, and we cite this figure in the relevant sentence as suggested by the reviewer (P9, L21).

14. P9, L22-26. This result is very surprising to me. If domperidone binds AcrB (as indicated by Fig. 6a and shown in ref 47), then why is it having an effect in a ΔacrB strain?

We agree that this is surprising. We speculate that the inhibitory effect of domperidone on the AcrAB-TolC multidrug efflux pump is not solely mediated by binding specifically to AcrB; the evidence for this in reference 47 is limited. We added a brief sentence to explain this point (P10, L23-25).

15. P10, L21-23. I'm not sure what this final sentence is doing, and it left me more confused. Do the authors consider this work a short evolution experiment? I'm assuming not, given that the authors appear to observe resistance gains approaching saturation. Even though (supposedly like a short evolution experiment) they don't observe diminishing returns epistasis. Are the authors saying that the fact that they didn't find it here is interesting, given it was a long enough experiment to detect it if it was present? If so, make this explicit.

We thank the reviewer for pointing out that this was unclear. Indeed, we wanted to emphasize that the absence of diminishing returns epistasis is notable because our experiments were conducted for an extended period and reached resistance levels approaching saturation, which suggests that it should have been detected if it were present. To clarify, we have rephrased the sentence in question and the conclusion of the paragraph (P11, L33-38). Note that we have also revised several of the previous lines in this and the previous paragraph (P11, L7-33), following comment 4 from Reviewer #2. We believe that these changes also contribute to clarifying the point raised here.

16. P10, L28-29. the authors statement that the populations converged on the same resistance mutations is not well justified, as they only looked at whether the same genes were mutated. Looking at they dataset, 94% of mutations only occurred in one population, and 3% only in two populations. If resistance mutations in most of the top gene targets are highly disruptive mutations, it makes sense they wouldn't see the exact same mutations. But this should be acknowledged and more careful word choice is needed to make clear the authors only look at the genotypic repeatability of evolution at an individual gene level, not an individual mutation level.

We rephrased these lines, as mentioned in the response to major comment 1c (this comment is almost identical).

17. P11, L12-14. So this sentence is saying, because the evolved reference population (with common mutations) often are not affected by knocking out the specific genes, then the effect of these genes on mutational paths often cannot be explained by epistasis? This sounds contradictory to the broader narrative that epistasis is responsible for the observed effects.

We understand how this may seem contradictory. We meant that the effect of these genes cannot be explained by direct epistasis between the gene deletion and the common resistance mutations (present in the evolved reference population). However, we speculate that epistasis, in a more general sense, could indirectly explain the effect. One possible mechanism for this could be epistasis between the deleted gene and an intermediate mutation. Although this intermediate mutation is not directly involved in the resistance mechanism, it is often necessary in the genetic background for the resistance mutation to fix. In other words, deleting a gene can hinder resistance evolution by disrupting intermediate steps in the mutational pathways toward resistance. We have changed the wording of this sentence to clarify that we meant direct epistasis with the resistance mutations, not epistasis in a general sense (P12, L34-46). Additionally, we have expanded the discussion of potential explanations for the observed phenomenon (P13, L6-11).

18. P11, L30-32. As I mentioned in minor comment 3, from Fig. 2f this really does not appear to be true for NIT. When that's 1/3 of the antibiotics tested, a blanket statement like this feels misleading.

Please see our response to minor comment 3 above. We now show in Fig. S4 that the trajectories of the UTI strains and the reference strain, *ΔlacA*, are relatively close to each other. We added an explanation of this (P5, L13-16). As mentioned in our response to minor comment 3, all results remain unchanged when all time points that reached the solubility limit are removed.

19. P14, L24. Typo: "...the general increasing general trend."

Corrected.

20. P15, L9-12. If AUC has to be used for MEC, then it should also be used for the other antibiotics. The values are not really comparable otherwise.

We primarily use dose-response curves to determine the IC_{50}. IC_{50} values are only compared between different strains (or time points) for the same antibiotic, i.e., we do not compare dose-response curves or IC_{50} values between different antibiotics. Therefore, whenever possible, we prefer to use the growth rate because it is a more robust and reproducible measure, and its absolute value is easily interpreted. Following the reviewer's suggestion, we confirmed that the IC_{50} values estimated using the AUC for NIT and TMP correlate almost perfectly with those estimated from growth rate (see plots below). We now briefly explain this in the Methods (P17, L23-28).

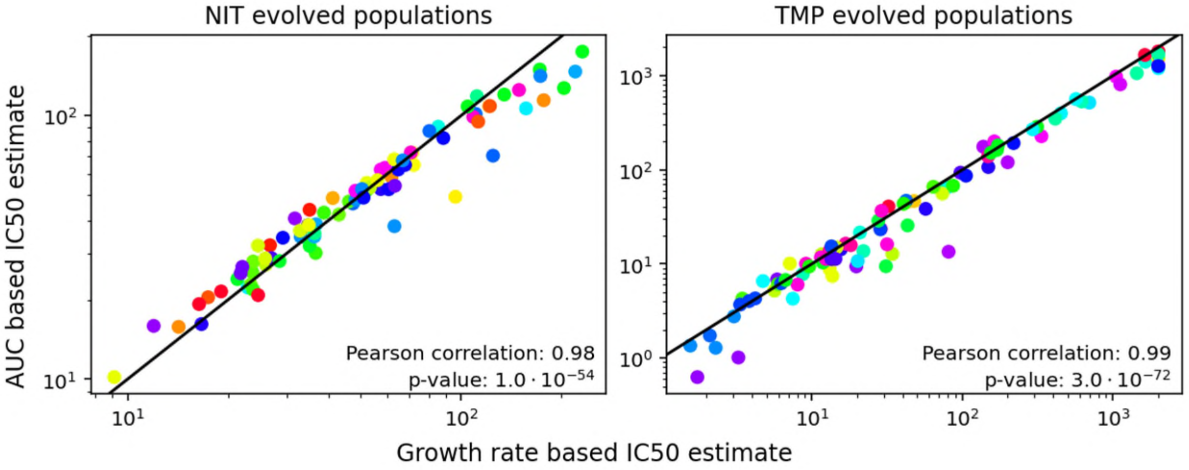

Fig. R2: IC_{50} estimates based on remeasured dose-response curves, with either AUC or growth rate as the response variable for NIT and TMP evolved populations. The black lines show the identity. Colors represent different gene-deletion strains.

21. P16, L22. Why is only data for NIT shown and not MEC or TMP?

We assume the reviewer is referring to Fig. S2, which is cited in those lines. We only added this technical control to show that the IC_{50} estimations directly obtained from our evolution experiments correlate with those estimated from remeasured dose-response curves. Thus, we did not consider it necessary to show this for all three antibiotics. However, we see the reviewer's point, and we have now added two panels to Fig. S2 showing data for TMP and MEC for the evolved strains. These strains exhibit resistance levels across the entire range relevant to our experiments.

22. Figure 1

a. for b-d caption: can you be explicit with what growth rate is normalized to. It's clear from methods, but I had to check what g/g0 meant.

b. Defining the population size as ($\propto$OD) and selection pressure as ($\propto$g/g0) confused me. This "$\propto$" proportion symbol isn't used elsewhere in the manuscript, so can you just say "is proportional to"?

c. for e-g caption: It looks like dark grey is 50% of data and light grey is 90% of the data?

Thanks. We have made all of the suggested changes to the legend of Fig. 1.

23. Table 1. There is no table legend. most columns are self-explanatory, but could benefit from an explanation of "steepness" and "maximum update factor". Also, what is exponent in the last row for this column? Also, column title says "Duration[days]" but units are given in hours.

We have added a legend to Table 1 that includes brief explanations of “steepness” and “maximum update factor”. The exponent in the last row of this column is the inverse of the steepness, indicating that a lower maximum update factor was used for antibiotics with higher steepness. We have included a comment about this in the legend. We have also corrected the units for the “Duration” column and added there additional information on the number of dilutions.

24. Issues with figure citations:

a. P4, L30. Cites Fig. 2b but the sentence is about NIT, which is Fig 2c

b. P4, L44. Fig S12 is cited after Fig S2

c. P5, L23-25. Fig 2h is cited after Figure 2i-j

d. P6, L17. Fig S7 is cited after Fig S3

e. P6, L36. Fig. 3b is not mentioned or referenced until later (after Fig 4)

f. P6, L47. I think you also want to cite Fig S4 here (not just Fig S5-S6)

g. P7, L46. You mean to reference Fig. 5a-c here, as all antibiotics are mentioned. But also, they're mentioned in text in different order from their a-b-c order in the figure.

h. P8, L5. Pretty sure you mean to cite Fig. 5f instead of Fig. 5c

i. P8, L8. Again, should be Fig. 5e. And really for consistency with L5 which cites Fig. S9, here should also cite the Fig. 3b multi-hit table.

j. P11, L12. Think this is supposed to cite Fig. 5e-f. Aren't Fig. 5a-c about all the deletions as a whole and not specific strains?

k. P11, L14. If I understand what you're trying to say, you want to cite: (Fig. 5g-h, Fig. S10)

l. The caption for Fig. S1a references the wrong methods section (should be the first one about choosing gene deletions, not second one about choosing strains to sequence.

We thank the reviewer for pointing out these issues, and we apologize for any confusion they may have caused. In the revised version, these issues have been corrected (see our response to major comment 5 above).

Regarding f.: we do not cite Fig S4 (now S11) here because the mutator strains did not clearly accelerate resistance evolution for NIT. We prefer not to discuss this observation in more detail because mutation rate effects are not the focus of our study (but that of many others) and we feel that it would be a distraction. However, we are following up on this interesting observation in a separate project.

Following g., we also changed the order of the panels a-f in Fig. 5 and the order in which the antibiotics are mentioned in the text to ensure consistency throughout the paragraph.

Point-by-point response to reviewers' comments

Reviewer #2 (Remarks to the Author):

I commend the authors for the substantial revisions made to the manuscript. The addition of new references, supporting figures (e.g., Fig. S1, Fig. S5), and expanded discussion has, in my view, addressed the major concerns raised during the first round of review by myself and the other reviewers. I now only have three minor comments, which I leave to the discretion of the authors and editor.

1) I appreciate the additional information on the functional categories of mutated genes provided in the revised Fig. 3. However, it appears that the diamond symbol is missing from the legend (envZ, ompR; presumably cell permeability?).

We thank the reviewer for pointing this out. We have added the definition of the diamond symbol (membrane transport) to the legend in Fig. 3.

2) On the same Fig. 3, regarding the newly added functional categories, I would suggest reordering the columns and introducing small gaps to divide the heatmap into seven column blocks (corresponding to the six functional categories with multiple hits plus a seventh "other targets" category) while preserving the current row order. This would make certain patterns more readily apparent. For instance, mutations in the efflux category appear to accumulate within the same genetic backgrounds (i.e., multiple hits per lineage), whereas mutations in other categories tend to be mutually exclusive (e.g., cell shape: mrdA vs. mreB; cell wall: mrcB, mepM, nlpI, slt, prc). While a simple statistical analysis may be sufficient to support this observation (e.g. Fisher's test), I believe this point is important, as it directly informs the patterns of epistasis among the mutations acquired by each background.

We thank the reviewer for their suggestion. While we agree with the reviewer that reordering the columns would help visualize certain patterns more clearly, we prefer to keep them in their current order. The current order follows the rank order of most-mutated genes, which aligns with the main message of this figure that fixed mutations are highly convergent at the gene level.

3) Finally, I appreciate that the authors now provide a more rigorous analysis showing that the distributions of separations between all gene-deletion backgrounds differ significantly from those within gene-deletion backgrounds. However, I am less convinced by the current presentation in Fig. S4. As it stands, the figure emphasizes that phenotypic variability is generally smaller than the typical resistance increase during evolution, which I don't think is that surprising. Instead, the more meaningful comparison to me is how trajectories differ within versus across genetic backgrounds. A shorter x-axis might help make this clearer. Additionally, providing a more intuitive number would make the result more tangible. For example, stating that "IC50 trajectories are on average X-fold more similar within backgrounds than across backgrounds". Getting an intuitive number like that was the intent of my original comment.

We thank the reviewer for their suggestion and for clarifying the intent of their original comment. However, we prefer to keep the scale of the x-axis in Fig. S4 as it is for the comparison. While a shorter x-axis would indeed stress the difference between within and across genetic backgrounds, as suggested by the reviewer, it would not allow for the comparison with the typical resistance increase. The current scaling allows the latter, and it also makes the difference between the lines showing within versus across genetic backgrounds visible.

Like I said, while I encourage the authors to address these final comments, I do not think any of them warrant an additional round of review from my side. I therefore congratulate the authors on this work and look forward to reading the final published version.
